# EEG alterations during wake and sleep in mild cognitive impairment and Alzheimer's disease

**DOI:** 10.1016/j.isci.2021.102386

**Published:** 2021-04-01

**Authors:** Aurora D'Atri, Serena Scarpelli, Maurizio Gorgoni, Ilaria Truglia, Giulia Lauri, Susanna Cordone, Michele Ferrara, Camillo Marra, Paolo Maria Rossini, Luigi De Gennaro

**Affiliations:** 1 Department of Psychology, University of Rome “Sapienza”, Via dei Marsi, 78, Rome 00185, Italy; 2 Department of Biotechnological and Applied Clinical Sciences, University of L'Aquila, Coppito (L'Aquila) 67100, Italy; 3 IRCCS Fondazione Santa Lucia, Rome, Italy; 4 Foundation Policlinico Universitario Agostino Gemelli IRCCS – Department of aging, neuroscience, orthopaedic and head-neck, Rome 00168, Italy; 5 Department of Neuroscience & Neurorehabil., IRCCS San Raffaele-Pisana, Rome, 00163, Italy

**Keywords:** Human Physiology, Cognitive Neuroscience, Chronobiology

## Abstract

Patients with Alzheimer's disease (AD) undergo a slowing of waking electroencephalographic (EEG) rhythms since prodromal stages, which could be ascribed to poor sleep quality. We examined the relationship between wake and sleep alterations by assessing EEG activity during sleep and (pre-sleep/post-sleep) wakefulness in AD, mild cognitive impairment (MCI) and healthy controls. AD and MCI show high sleep latency and less slow-wave sleep. Reduced sigma activity characterizes non-rapid eye movement (NREM) sleep, reflecting sleep spindles loss. The EEG slowing characterizes REM sleep and wakefulness of AD and MCI, with strong correlations among the two phenomena suggesting common neuropathological mechanisms. Evening-to-morning variations in waking EEG revealed the gradual disappearance in MCI and AD of overnight changes in delta activity, indicating a progressive decay of sleep restorative functions on diurnal activity that correlates with the impairment of sleep high-frequency activity in AD. Our findings support a linkage between wake and sleep alterations, and the importance of sleep-related processes in Alzheimer's disease progression.

## Highlights

• The EEG slowing characterizes wake and REM sleep in AD and MCI compared to controls
• NREM sleep reveals a posterior reduction of sigma EEG power compared to controls
• MCI and AD show a progressive decay of sleep restorative functions on diurnal EEG
• EEG slowing in REM sleep shows the highest correlation with cognitive impairment

## Graphical abstract

**Figure:**
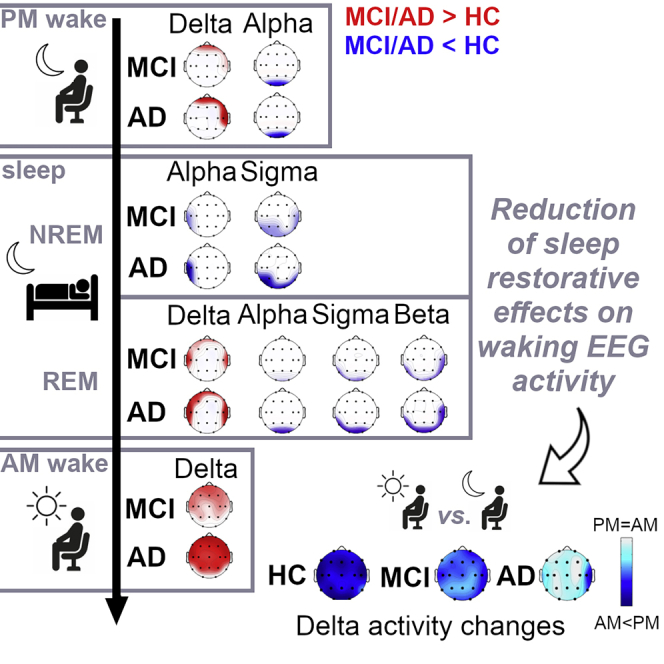

## Introduction

Alzheimer's disease (AD) is a neurodegenerative syndrome representing the most common cause of dementia in the elderly population. The electroencephalography (EEG) allows detecting changes in cortical activity associated with AD, even at early stages. The hallmark of the resting state EEG in patients with AD is the slowing of cortical rhythms, consisting of increased low-frequency (0.5-7.0 Hz) and decreased high-frequency activity (Babiloni et al., 2015; Jeong, 2004). Similar EEG features affect mild cognitive impairment (MCI) subjects, a condition being prodromal to AD in more than half of cases (Babiloni et al., 2006; Galluzzi et al., 2001; Petersen et al., 2001; Scheltens et al., 2002). The EEG slowing correlates with the functional, structural, and cognitive changes in the disease progression (Babiloni et al., 2006; Claus et al., 2000; Jelic et al., 1996) and has been considered an EEG expression of the neurodegenerative process (Dringenberg, 2000).

EEG activity during sleep is also affected in MCI and AD. Recent studies reported a significant reduction of sleep spindles (Gorgoni et al., 2016) and K-complexes (De Gennaro et al., 2017) during non-rapid eye movement (NREM) sleep in patients with AD and MCI. Instead, preliminary observations in REM sleep suggested an increase of low-frequency rhythms paralleled by the reduction of high-frequencies, mirroring those occurring in the waking EEG (Brayet et al., 2016; Hassainia et al., 1997). It is worth noting that local sleep EEG oscillations have a crucial role in learning processes and plastic mechanisms. In particular, several electrophysiological hallmarks of both NREM (i.e., slow waves, sleep spindles, hippocampal ripples) and REM sleep (i.e., theta activity) are actively involved in memory consolidation (Klinzing et al., 2019). Starting from this evidence, the assessment of local sleep alterations and their functional meaning has an essential clinical relevance in the field of neurodegenerative disorders. Interestingly, recent findings suggest that the alteration of sleep electrophysiology could be related to the cognitive status of AD patients and MCI subjects. For instance, both reduced sleep spindle and K-complex density appear associated with the degree of cognitive decline (De Gennaro et al., 2017; Gorgoni et al., 2016; Reda et al., 2017)

The pattern characterizing the cortical activity in these patients mimics the effect of the sleep deprivation in healthy subjects (Curcio et al., 2003; Gorgoni et al., 2014; Marzano et al., 2010), suggesting that it could be—at least in part—a direct consequence of their poor sleep quality. In other words, the increase of slow-frequency cortical activity during wake could also reflect a robust drive to sleep, linked to a dysfunction of sleep-related homeostatic regulation processes. Some studies support the notion of a link between the progression of neurodegenerative phenomena and the impairment of the sleep-wake cycle (Cordone et al., 2019; Lim et al., 2014). According to this view, the early deposit of the amyloid plaques in specific brain regions which is typical of AD could interfere with the sleep-wake cycle regulation, resulting in sleep fragmentation and a reduction of slow-wave sleep (SWS). On the other hand, a good sleep quality seems to play a protective role against the amyloid accumulation: β-amyloid levels increase with time awake in mice, while NREM sleep predicts the clearance of β-amyloid (Kang et al., 2009; Xie et al., 2013). Signs of sleep disruptions are associated with AD biomarkers in humans (Lucey et al., 2019; Mander et al., 2015; Winer et al., 2019) and animals (Holth et al., 2017; Menkes-Caspi et al., 2015). Sleep deprivation and selective SWS disruption enhance levels of β-amyloid (Ju et al., 2017; Lucey et al., 2018; Ooms et al., 2014), tau and tau spreading (Holth et al., 2019). Finally, longitudinal studies suggest that sleep disruption is associated with AD-related outcomes (Lim et al., 2013; Winer et al., 2020). Therefore, sleep disturbances, increasing the time spent in wakefulness, might negatively contribute to the AD condition.

Surprisingly, no study investigated the relationship between EEG characteristics during wakefulness and sleep in AD patients. Hence, we recorded the EEG in a large cohort of AD, MCI, and heathy control (HC) subjects during wakefulness and sleep in order to describe the topographic changes across the wake-sleep cycle, specifically during NREM and REM sleep, and during wakefulness in the pre-sleep evening and post-sleep morning. Such investigation was aimed to determine whether any of the evaluated EEG characteristics (or a combination of them) would be useful in discriminating AD, MCI, and HC. Starting from the reported findings of altered electrophysiology during wakefulness, NREM, and REM sleep associated with the AD pathology, we expected to observe (a) state- and frequency-specific topographical EEG patterns in AD, MCI, and HC, and (b) a relation between the main local EEG alterations characterizing the clinical samples and the degree of cognitive decline. With the aim to assess the hypothesis that the increased slow-frequency activity during wakefulness in AD/MCI could partially represent a sign of enhanced sleep drive, we also evaluated whether sleep-related homeostatic factors differently modulate the waking EEG activity in these groups. Finally, since EEG slowing has been observed in both wakefulness and REM sleep in AD and MCI, we hypothesized the existence of a common neuropathological mechanism underling these phenomena. For this reason, we evaluated the correlation among EEG slowing during wakefulness and the corresponding phenomenon during REM sleep.

## Results

### EEG alterations in MCI and AD across the wake-sleep cycle

#### Sleep macrostructure

Unexpectedly, no difference between groups was present for any of the explored parameters of sleep macrostructure, except for sleep onset latency and the SWS duration (Table 1). Specifically, the AD and MCI groups needed significantly more time to fall asleep and spent significantly less time in SWS than the HC group.

**Table 1:** Sleep macrostructure in AD, MCI and HC groups

	AD	MCI	HC		AD *vs* MCI	AD *vs* HC	MCI *vs* HC
	*Mean*	*Mean*	*Mean*	*F*_*2,147*_	*t*_*98*_	*t*_*98*_	*t*_*98*_
	*(SD)*	*(SD)*	*(SD)*	(p)	(p)	(p)	(p)
**Sleep macrostructural variable**							

**SO latency *min***	35.86	23.89	16.52	**6.02**	1.84	**3.01**	**2.16**
	(42.32)	(17.76)	(16.35)	(**0.003**)	(0.07)	(**0.003**)	(**0.03**)
**REM latency *min***	110.20	117.93	104.86	0.35			
	(80.74)	(89.19)	(59.77)	(0.70)			
**WASO *min***	82.63	88.45	78.94	0.46			
	(50.75)	(51.58)	(47.71)	(0.63)			
**N1*%***	9.94	8.73	6.62	2.73			
	(9.46)	(6.25)	(5.12)	(0.07)			
**N2*%***	74.28	74.44	76.54	1.14			
	(9.52)	(9.20)	(5.86)	0.32			
**N3*%***	0.20	0.12	0.65	**7.31**	1.08	**−2.49**	**−3.13**
	(0.48)	(0.23)	(1.16)	(**0.0009**)	(0.28)	(**0.01**)	(**0.002**)
**REM *%***	15.58	16.71	16.19	0.28			
	(9.16)	(7.67)	(5.56)	0.76			
**TBT *min***	389.05	411.07	385.47	2.11			
	(58.19)	(91.02)	(44.72)	0.12			
**TST *min***	270.09	284.52	290.72	1.20			
	(76.30)	(67.30)	(60.32)	0.30			
**SEI %**	69.27	70.38	75.51	2.47			
	(15.78)	(15.79)	(13.23)	0.09			
**ISA #**	18.98	19.18	20.2	0.21			
	(11.45)	(9.45)	(9.44)	0.81			

Mean and standard deviation (SD) of the polysomnographic variables of AD, MCI, and HC groups. The results of the one-way ANOVAs (*F* and p values) are also reported, with *post hoc* unpaired t test (*t* and p values) when ANOVAs were significant (p ≤ 0.05). Significant between-groups differences are indicated in bold.

AD, Alzheimer's disease; HC, healthy controls; ISA, intra-sleep awakenings; MCI, mild cognitive impairment; REM, rapid eye movement; N1, NREM 1 stage; N2, NREM 2 stage; N3, NREM 3 stage; SD, standard deviation; SEI, sleep efficiency index; SO, sleep onset; TBT, total bed time; TST, total sleep time; WASO, wake after sleep onset.

#### EEG power in NREM sleep

**Figure 1:**
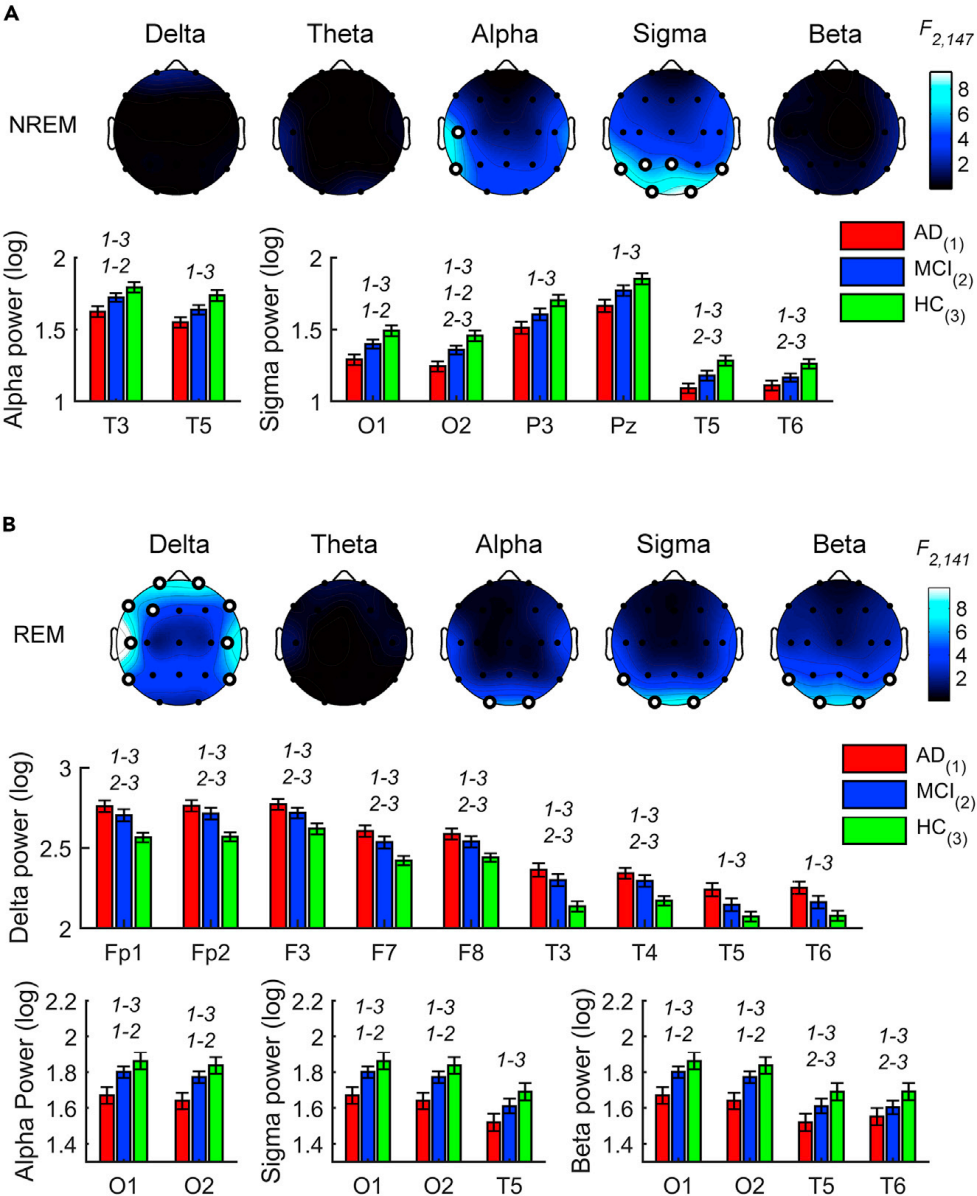
Sleep EEG alterations in AD, MCI, and HC groups Statistical maps (F-values) of the one-way ANOVAs (AD *vs*. MCI *vs*. HC) on the spectral powers during NREM (A) and REM (B) sleep and histograms of spectral power (mean ± SEM) in AD (red), MCI (blue) and HC (green) groups at the cortical sites and frequency bands showing a significant between-groups difference in the one-way ANOVAs. Maps are scaled between minimal and maximal F-values across the statistical comparisons in all frequency bands. White dots represent significant statistical differences, according to the FDR correction (p ≤ 0.0102). *y* axis of histograms has non-zero origin to magnify standard errors visibility. The groups with significant differences in the post hoc pairwise comparisons by two-tailed unpaired t test (p ≤ 0.05) are reported by numerical code (1: AD, 2: MCI, 3: HC). See also Figure S1 and Table S1.

The EEG activity during NREM sleep in the three groups (N_AD_: 50, N_MCI_, 50; N_HC_, 50; Figure S1A) showed significant between-group differences for the alpha band on the left temporal cortex and the sigma band in the posterior temporo-parieto-occipital sites (F_2,147_ ≥ 5.35, p ≤ 0.0058; Figure 1A and Table S1). Post hoc comparisons revealed that AD participants showed lower alpha activity than MCI and HC. Similarly, the AD group reported lower EEG activity than the HC group in the sigma frequency band. The sigma activity was also decreased in AD compared to the MCI group at the occipital sites and in MCI compared to the HC group at the temporo-occipital areas.

#### EEG power in REM sleep

The EEG activity during REM sleep in the three groups (N_AD_: 46, N_MCI_: 49, N_HC_: 49; Figure S2B) revealed differences in the delta activity at the frontotemporal regions and at the occipital and temporo-occipital regions for the alpha, sigma, and beta power (F_2,141_ ≥ 5.04, p ≤ 0.0077; Figure 1B and Table S1). Post hoc t-tests indicated that the differences reflect the slowing of the EEG in the clinical samples, showing higher EEG activity than HC in the delta frequency band and lower power at the higher frequencies. Specifically, the AD group reported higher delta activity than the HC in a large cluster of frontotemporal recording sites, while no differences between AD and MCI groups were significant. Similarly, the MCI group also reported higher frontotemporal delta power than the HC. The AD group showed lower EEG activity in the high-frequency bands than the HC and the MCI group at the occipital-temporal sites. In the beta frequency band, the MCI group also showed significantly lower EEG activity than the HC group over T5 and T6.

#### Waking EEG in the evening

**Figure 2:**
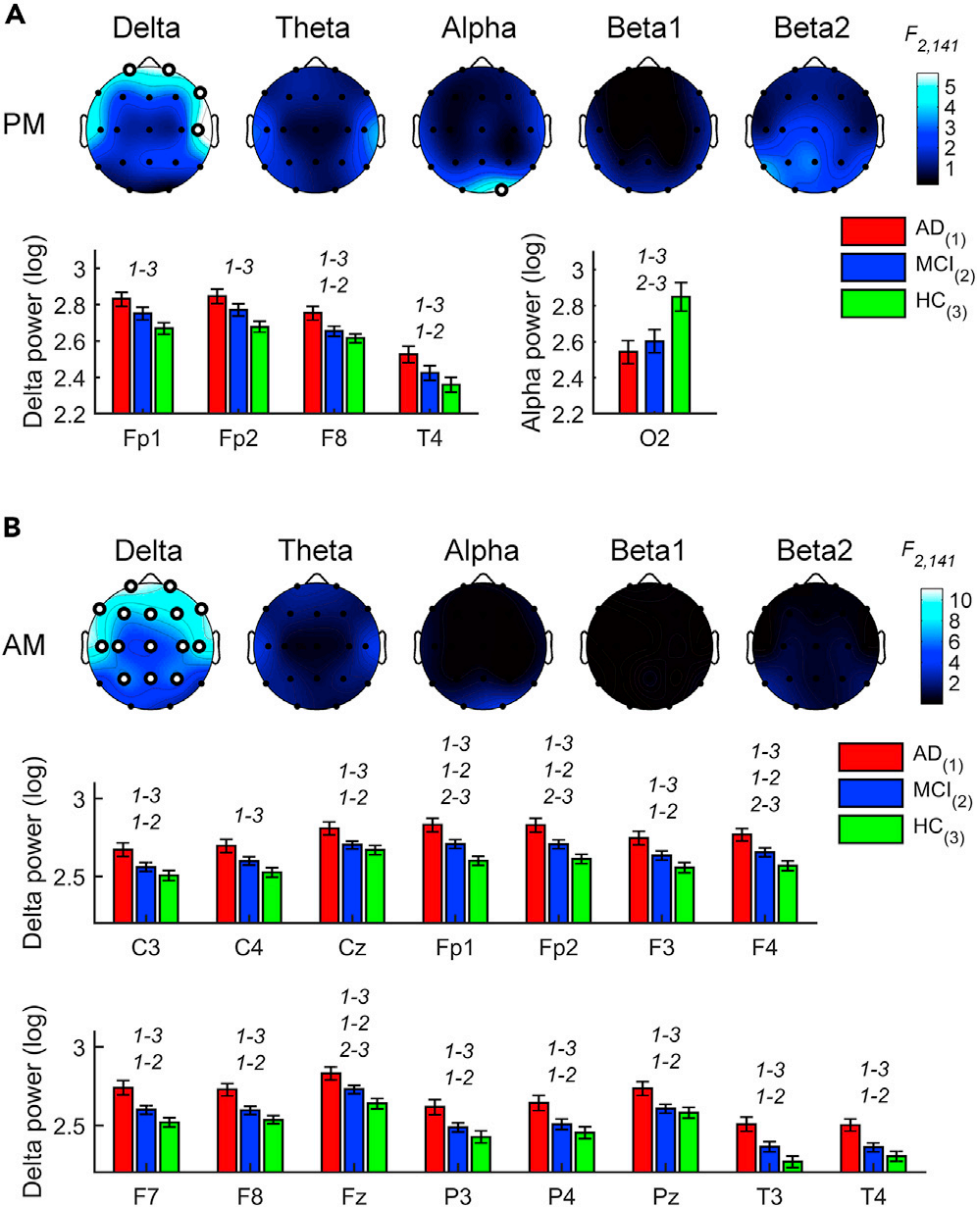
EEG alterations during evening and morning wakefulness in AD, MCI, and HC groups Statistical maps (F-values) of the one-way ANOVAs (AD *vs*. MCI *vs*. HC) on the spectral powers during evening (A) and morning (B) wakefulness and histograms of spectral power (mean ± SEM) in AD (red), MCI (blue) and HC (green) groups at the cortical sites and frequency bands showing a significant between-groups difference in the one-way ANOVAs. Maps are scaled between minimal and maximal F-values across the statistical comparisons in all frequency bands. White dots represent significant statistical differences, according to the FDR correction (p ≤ 0.0102). *y* axis of histograms has non-zero origin to magnify standard errors visibility. The groups with significant differences in the post hoc pairwise comparisons by two-tailed unpaired t test (p ≤ 0.05) are reported by numerical code (1: AD, 2: MCI, 3: HC). See also Figure S2 and Table S2.

The waking EEG activity recorded in the evening hours (N_AD_: 45, N_MCI_: 49, N_HC_: 50; Figure S2A) displayed significant differences at the prefrontal and right frontotemporal sites for the delta band and at the right occipital derivation only for the alpha band (F_2,141_ ≥ 5.06, p ≤ 0.0075, Figure 2A and Table S2). Prefrontal delta power was significantly higher in the AD compared to the HC group, while the right frontotemporal delta activity increased in the AD compared to both the HC and MCI groups. As expected, the occipital alpha power was reduced in the AD and MCI groups compared to the HC group.

#### Waking EEG in the morning

In the morning EEG, the three groups (N_AD_: 44; N_MCI_: 49; N_HC_: 50; Figure S2B) showed differences only in the delta band (F_2,140_ ≥ 4.74, p ≤ 0.0102; Figure 2B and Table S2), with a prevalence of the delta activity in AD compared to both HC and MCI groups. On the other hand, the EEG activity of the MCI group differed from that of HC only at the frontal sites.

### Changes in the waking EEG after a night of sleep

**Figure 3:**
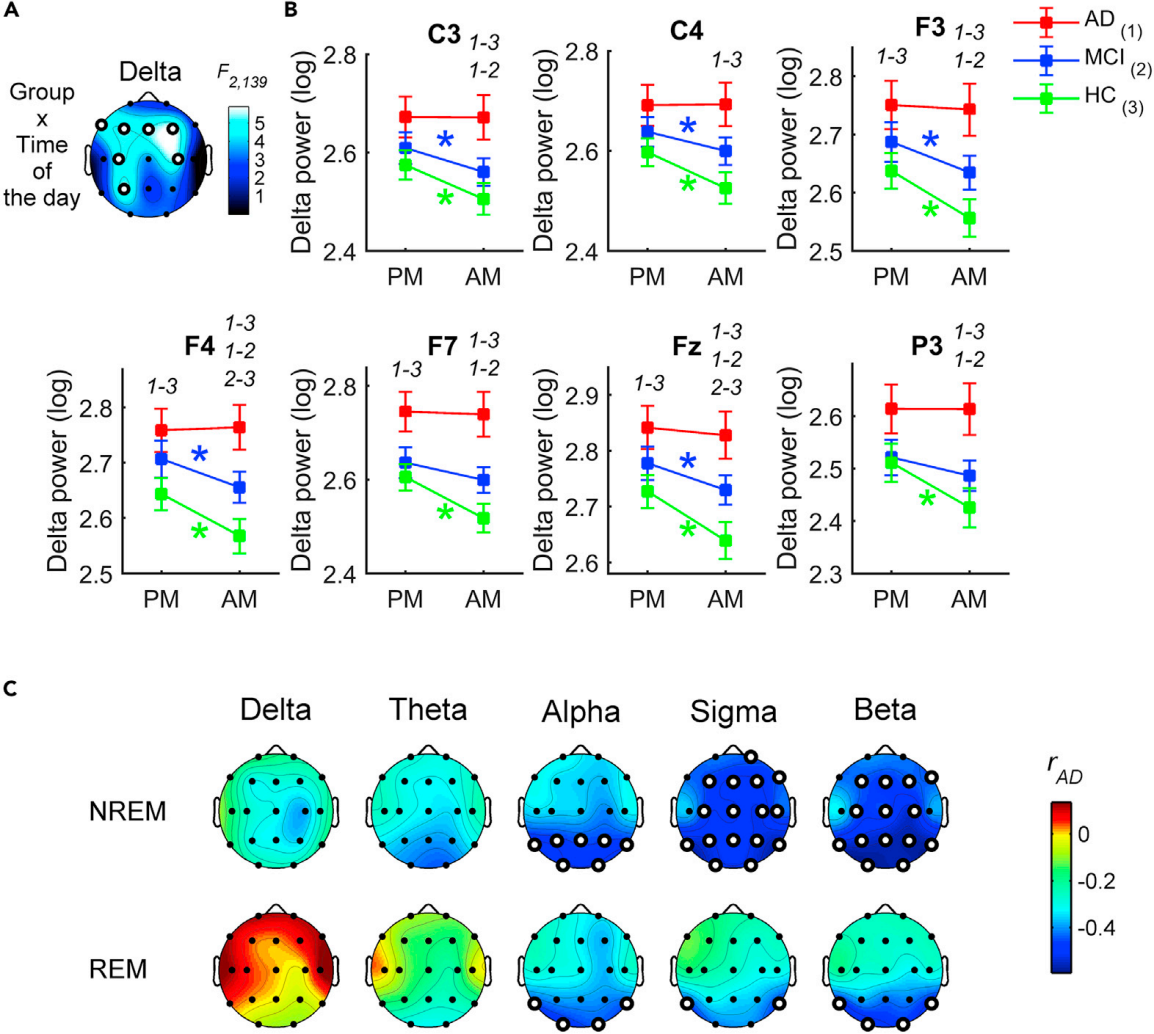
Changes in the waking EEG delta power across a night of sleep in AD, MCI, and HC groups and its relationship with the sleep activity in the AD group (A) Statistical map (F-values) of the *Group* x *Time of day* interaction of the mixed-design ANOVAs [between-subjects factor *Group*: AD vs. MCI vs. HC; within-subject factor *Time of day*: evening wakefulness (PM) vs. evening wakefulness (AM)] on spectral power in the delta band. White dots represent significant statistical interactions, according to the FDR correction (p ≤ 0.0102). See also Table S3. (B) Spectral power (mean ± SEM) in AD (red), MCI (blue) and HC (green) groups during evening (PM) and morning (AM) wakefulness at the cortical sites showing a significant *Group* x *Time of day* interaction in the mixed-design ANOVAs. Colored asterisks represent significant PM *vs*. AM differences (two-tailed paired t-tests, p ≤ 0.05) in the specific group coded by the asterisk color (red ∗: AD, blue ∗: MCI, green ∗: HC), while the groups with significant differences in the pairwise between-group comparisons (two-tailed unpaired t-tests, p ≤ 0.05) are reported by numerical code (1: AD, 2: MCI, 3: HC). (C) Statistical maps of the (two-sided) Pearson's *r* correlation coefficients between changes in waking EEG delta power before and after sleep at a representative frontal site (F4) and the EEG spectral power during NREM sleep (first row) and during REM sleep (second row) in AD. The maps are scaled according to minimal and maximal *r*-values across all frequency bands and sleep stages. White dots represent significant correlations, according the FDR correction (p ≤ 0.0054). MCI and HC did not show significant correlations. See also Table S4.

We also investigated if sleep-related homeostatic factors modulate between-group differences in waking cortical activity since the two resting state recordings were performed before and after sleep. We found significant *Group* x *Time of day* interactions in the waking EEG (N_AD_: 43; N_MCI_: 49; N_HC_: 50) for the delta band at bilateral frontal and central regions, and the left parietal area (F_2,139_ ≥ 4.76, p ≤ 0.0102; Figure 3A and Table S3). The post hoc t-tests comparing the AM *vs*. PM (Figure 3B) showed that sleep ‘restored’ cortical activity for MCI and HC groups by inducing a significant decrease of the delta frequency band in the morning EEG. Notably, these comparisons were not significant in the AD group.

### Relationships between changes in waking activity after sleep and activity during sleep

To further clarify the possible relations between wake and sleep EEG activity in the AD, MCI, and HC groups (N_AD_: 39; N_MCI_: 48; N_HC_: 49), we correlated the overnight changes of waking EEG in the delta band with the whole EEG activity during sleep. We considered the F4 site as the most representative channel for the changes in the delta band (i.e., it exhibits the largest *Group* x *Time of day* interaction). In the AD group, the correlations (Figure 3C and Table S4) revealed that changes in waking EEG are strongly related to the nocturnal activity during the NREM sleep and REM sleep (r ≤ −0.44, p ≤ 0.0050). Specifically, the frontal changes of the delta waking activity negatively correlated with the temporo-parieto-occipital alpha activity and with the sigma and beta activity mostly in posterior areas during NREM sleep. Similarly, the correlation was significant with the temporal sigma activity and with temporo-occipital alpha and beta activity of the REM sleep. Indeed, the lesser the patients expressed high-frequency EEG rhythms during sleep, the more the delta activity in the morning was similar to that in the evening. A similar correlations pattern has been found by considering the other cortical sites with a significant *Group* x *Time of day* interaction (data not shown). Conversely, this relation was not present in the MCI and HC groups.

### The EEG slowing during REM sleep and wakefulness

**Figure 4:**
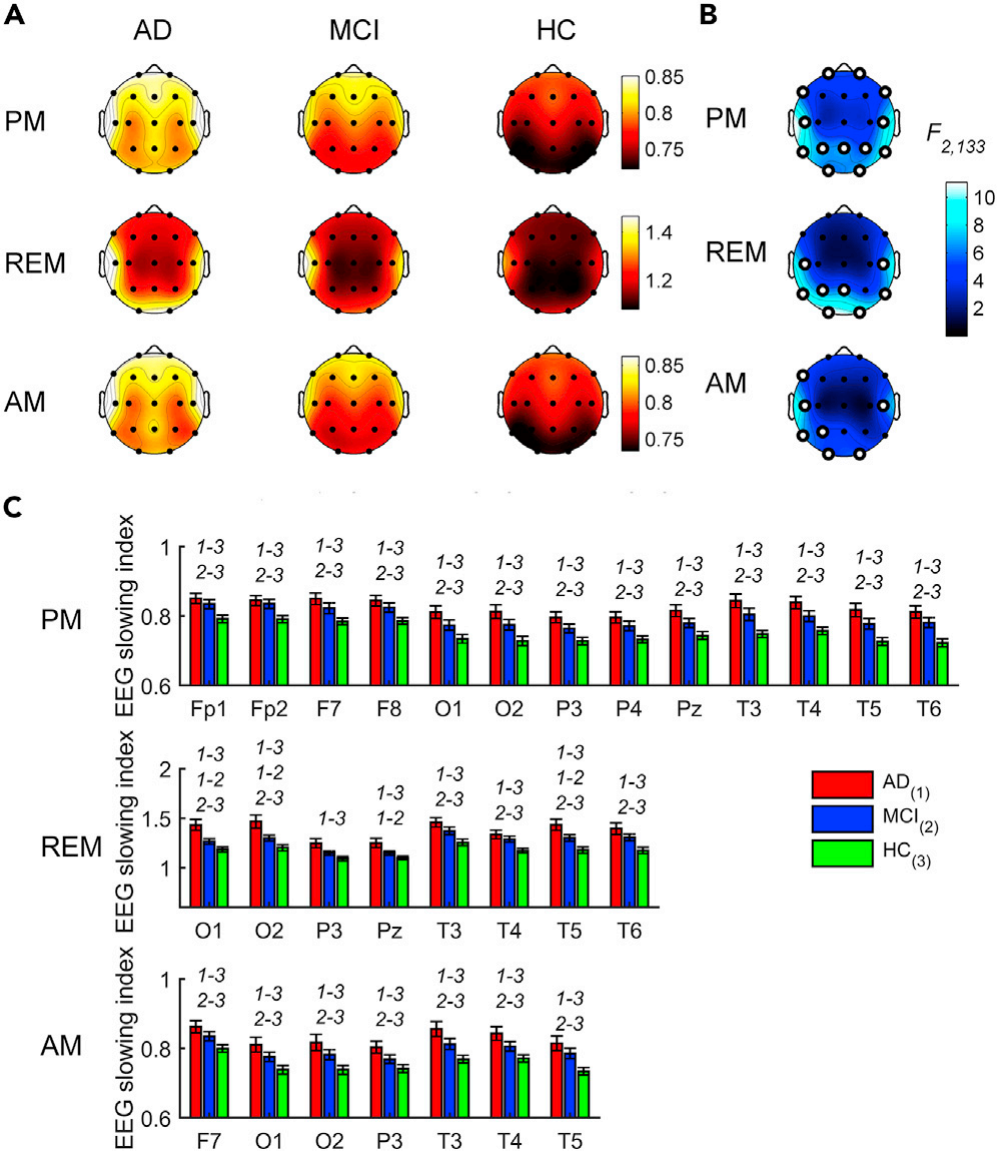
The EEG slowing during REM sleep and wakefulness in AD, MCI, and HC groups (A) Topographic maps of the EEG slowing index [(delta + theta)/(alpha + sigma + beta)] during evening wakefulness (PM, first row), REM sleep (second row), and morning wakefulness (AM, third row) in AD (first column), MCI (second column) and HC (third column) groups. The topographic maps are scaled between minimal and maximal values of the three groups within each condition. (B) Statistical maps (F-values) of the one-way ANOVAs (AD *vs*. MCI *vs*. HC) on the EEG slowing index in each condition. Maps are scaled between minimal and maximal F-values across the statistical comparisons in all conditions. White dots represent significant statistical differences, according to the FDR correction (p ≤ 0.0102). See also Table S5. (C) Histograms of the EEG slowing index (mean ± SEM) in AD (red), MCI (blue) and HC (green) groups at the cortical sites showing a significant between-groups difference in the one-way ANOVAs for each condition. *y* axis of histograms has non-zero origin to magnify standard errors visibility. The groups with significant differences in the post hoc pairwise comparisons by two-tailed unpaired t test (p ≤ 0.05) are reported by numerical code (1: AD, 2: MCI, 3: HC).

Since a slowing of the EEG characterized both wakefulness and REM sleep, we asked how these two phenomena are associated across different scalp locations (N_AD_: 39; N_MCI_: 48; N_HC_: 49). The synthetic EEG slowing index showed a temporo-occipital maximum during REM sleep, while a temporo-frontal prevalence with a middle-frontal peak characterized the EEG slowing during wakefulness (Figure 4A).

The comparisons (Figure 4B and Table S5) revealed that the groups differed at all lateral, frontopolar, and parietal areas during evening wakefulness (F_2,133_ ≥ 5.16, p ≤ 0.0069). The evening EEG slowing was significantly higher in AD and MCI than in HC, while it did not differ between AD and MCI (Figure 4C). REM sleep was associated with significant differences at the temporo-occipital and parietal areas (F_2,133_ ≥ 6.09, p ≤ 0.0029). AD showed significantly greater EEG slowing than HC in these areas, and MCI higher EEG slowing than HC in the temporo-occipital but not in the parietal regions. The REM EEG slowing significantly differed between AD and MCI over the most posterior sites.

In the morning, the EEG slowing showed smaller between-group differences than the other conditions (F_2,133_ ≥ 4.97, p ≤ 0.0083) but confirmed the temporal and occipital regions as the most affected areas. As in the evening, the EEG slowing in the morning was comparable in AD and MCI, while it was higher in AD than HC at all cortical locations significant at the *omnibus* ANOVAs (t_86_ ≥ 2.99, p ≤ 0.0036) and at all sites but P3 in the MCI *vs*. HC comparison (t_95_ ≥ 1.99, p ≤ 0.05).

**Figure 5:**
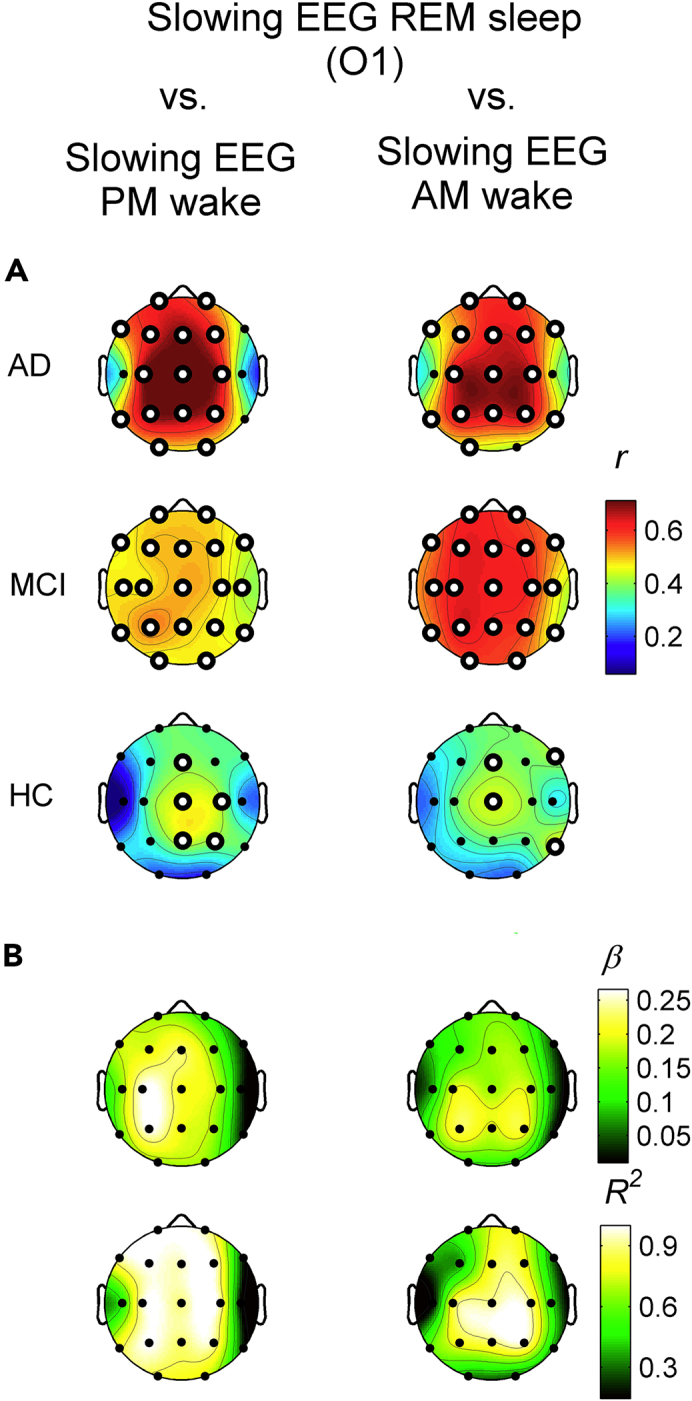
Correlation between the EEG slowing in REM sleep and the EEG slowing in morning and evening wakefulness (A) Statistical maps of the Pearson's *r* correlation coefficients between the EEG slowing index during REM sleep at one representative occipital site (O1) and the EEG slowing during evening (PM, first column) and morning (AM, second column) wakefulness in AD (first row), MCI (second row) and HC (third row) groups. Maps are scaled between minimal and maximal *r*-values across the conditions and groups. White dots represent significant correlations, according to the FDR correction (p ≤ 0.0054). See also Table S6. (B) Topographic maps of the angular coefficients (β, first row) and R^2^ (second row) of the linear regression fits computed on the (z-transformed) correlation coefficients in the three groups (for graphic purposes only) for the evening (first column) and morning (second column) resting state conditions. Maps are scaled between minimal and maximal values across the conditions.

The EEG slowing at cortical location showing the most robust between-group difference during REM sleep, i.e. O1 (F_2,133_ = 11.10, p = 0.000035), has been correlated with the topography of the EEG slowing during evening and morning wakefulness, separately for each group. The magnitude of these wake-REM correlations linearly increased from HC to AD (Figure 5A and Table S6). In the HC group, the highest significant correlations were those with the EEG slowing at Cz (*r* = 0.45, p = 0.0013) and Pz (*r* = 0.44, p = 0.0014) for the evening EEG, and at Cz (*r* = 0.44, p = 0.0016) and T6 (*r* = 0.45, p = 0.0012) for the morning EEG.

The MCI EEG slowing in the evening wakefulness showed higher correlations than HC for all cortical sites (*r* ≥ 0.44, p ≤ 0.0019), but T4. In the morning, it was even more strongly correlated with the REM slowing and showed correlations higher than 0.50 in all cortical sites (*r* ≥ 0.52, p ≤ 0.00013) but T4 and T6, where the correlations were still significant. The maximal correlation with the REM EEG slowing in the MCI group has been found at P3 for both the evening (*r* = 0.54, p = 0.00007) and the morning (*r* = 0.64, p = 8.3 × 10^−7^) recordings.

Finally, the AD group reported the highest correlations between evening wake and REM sleep EEG slowing compared to the MCI and HC groups, at least in central, frontal, and parietal areas (Cz, Fz, P3: *r* ≥ 0.70, p ≤ 6.9 × 10^−7^). The morning EEG slowing in AD showed slightly lower, but still very robust, correlations than the morning one, with a centro-parietal prevalence (C3, C4, Cz, P3, P4, Pz: 0.64 ≤ *r* ≤ 0.67, 3.8 × 10^−6^ ≤ p ≤ 0.000010). As in the MCI, the EEG slowing at the temporal regions during both the evening and morning wakefulness was weakly or not correlated to the REM EEG slowing.

With a merely descriptive purpose, this across-group linear increase of the wake-REM correlations has been quantified by computing the angular coefficients and the corresponding R^2^ values (Figure 5B). The EEG slowing during evening wakefulness of a cluster of bilateral parietal, central, and frontal recording sites progressively showed larger correlations from HC to AD. During morning wakefulness, this trend involved parietal and central areas.

### Correlation between EEG alterations and cognitive impairment

**Figure 6:**
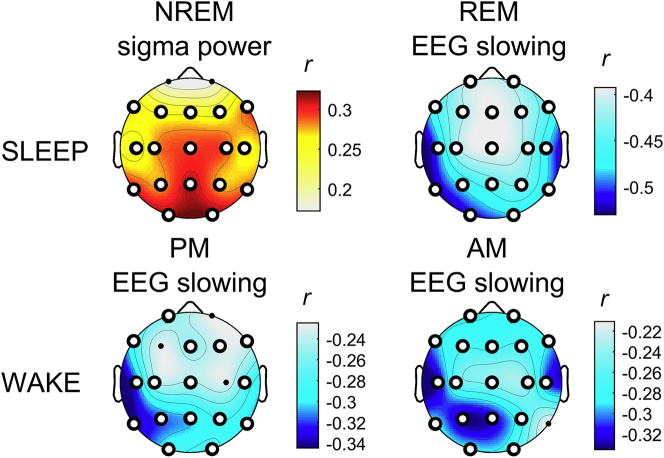
Correlation between the main EEG alterations in AD and MCI and cognitive impairment Statistical maps of the (two-sided) Pearson's *r* correlation coefficients between the main EEG alterations during sleep (i.e. NREM sleep sigma power and EEG slowing during REM, first row) and wakefulness (evening end morning EEG slowing, second row) and the MMSE scores. The maps are scaled according to the minimal and maximal *r*-values across the conditions. White dots represent significant correlations, according the FDR correction (p ≤ 0.0054). See also Table S7.

The most distinctive characteristics of our clinical samples (i.e., sleep onset latency and SWS duration (%), EEG slowing of wake and REM sleep and the decreased NREM sleep sigma activity) have been correlated to the cognitive decline, as measured by the MMSE scores (Figure 6 and Table S7). Macrostructural sleep alterations showed only a weak association with the cognitive status (sleep onset latency: rho = −0.20, p = 0.012; SWS%: rho = 0.14, p = 0.092) compared to other EEG indexes. Regardless of the condition, the EEG slowing index was significantly (negatively) correlated to the MMSE scores (*r* ≤ −0.23, p ≤ 0.0054). Specifically, greater EEG slowing was associated with worse cognitive impairment, as indicated by lower MMSE scores. The correlation was higher for REM sleep EEG alterations (−0.52 ≤ *r* ≤ −0.39; 3.51 × 10^−11^ ≤ p ≤ 1.15 × 10^−6^) than wakefulness (−0.34 ≤ *r* ≤ −0.23, 0.000023 ≤ p ≤ 0.0054) at all scalp recording sites. Sigma power during NREM sleep was also significantly correlated to the cognitive impairment but in the opposite direction, with lower sigma power linked to lower MMSE scores. The magnitude of the correlations between MMSE scores and NREM sigma power (0.24 ≥ *r* ≥ 0.32; 0.000055 ≤ p ≤ 0.0027) was comparable to that with EEG slowing during the wake but lower than the correlation with the EEG slowing during REM sleep.

## Discussion

The study describes the complex pattern of topographic and frequency-specific changes in the EEG activity of AD and MCI compared to HC across different behavioral states [evening wake, sleep (NREM and REM), and morning wake], also investigating their relationship.

**Figure 7:**
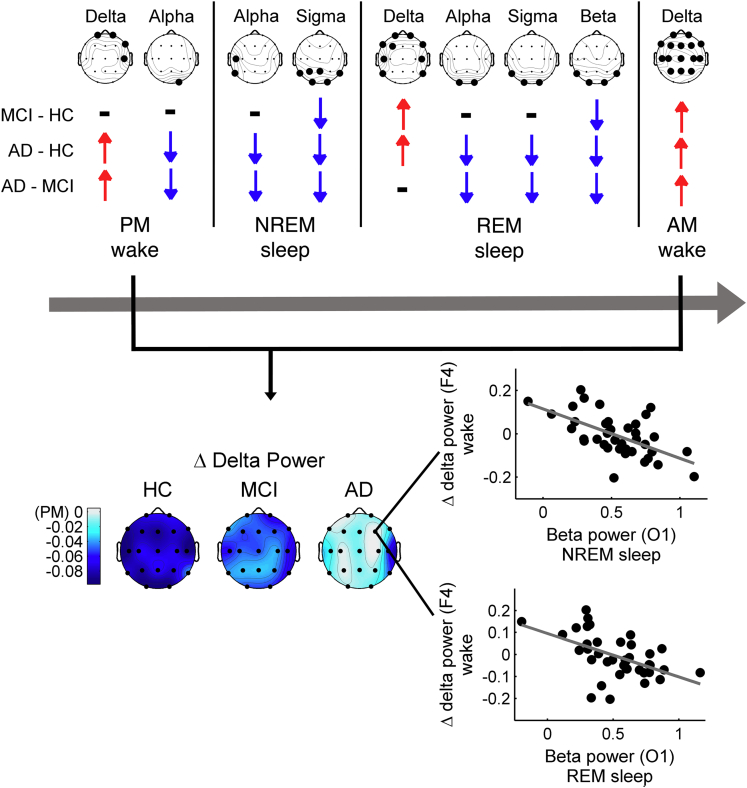
Summary of the topographic and frequency-specific EEG features of cortical activity during wakefulness and sleep in MCI and AD Topography of the frequency-specific significant differences in cortical activity in MCI and AD as compared to HC (one-way ANOVAs, p ≤ 0.0102) during evening wakefulness, NREM and REM sleep, and morning wakefulness (upper). The direction of the difference in the pairwise comparisons is given by the red and blue arrows representing significant increased and decreased cortical activity (two-tails unpaired t-tests, p ≤ 0.05) in MCI and AD compared to HC and in AD compared to MCI, respectively. The gradual disappearance of the changes in delta power between pre-sleep and post-sleep wakefulness EEG from HC to AD condition (bottom) is also shown. The maps represent AM log_10_(Delta power) – PM log_10_(Delta power) differences for HC, MCI, and AD groups. The negative values of the blue scale indicates that delta power decreases after sleep. The scatterplots show the linear correlation among this delta power change at a frontal representative site (F4) and the high-frequency activity during NREM and REM sleep at a posterior representative site (O1) in the AD group. F4 and O1 derivations were respectively chosen as representative for delta power changes in waking EEG and posterior beta power activity during sleep since they showed the highest correlation in the analysis reported in Figure 3C.

In synthesis (Figure 7), we found that the main EEG indexes differentiating AD and MCI from HC involve the temporo-parieto-occipital decrease of the alpha and sigma EEG activity during both NREM and REM sleep, also affecting the beta band in the latter case, and the temporo-frontal increase of the delta activity during REM sleep and wakefulness. We also showed that waking cortical activity undergoes only small changes after sleep in these clinical populations compared to HC. The EEG modifications during wakefulness and sleep are mutually correlated and they correlate with the degree of cognitive impairment, with the REM EEG slowing showing the strongest association.

### Macrostructural sleep changes

Although a large body of evidence points to a worsening of sleep macrostructure in AD (Peter-Derex et al., 2015; Petit et al., 2004) and MCI (D'Rozario et al., 2020), representing an exacerbation of the sleep disruption observed in the healthy elderly (Peter-Derex et al., 2015), our findings only partially replicate such evidence. We found an increased sleep onset latency and a decreased SWS duration in both AD and MCI groups, confirming an altered sleep macrostructure, but our clinical samples exhibited more preserved sleep quality than expected. However, the specific pattern of macrostructural disruption in AD and MCI participants appears variable across studies. Beyond possible methodological and demographic sources of variability, sleep changes in this population appear strictly linked with the progression of the disease but such relationship may not necessarily be linear (Villa et al., 2015). In light of our large sample size, the observation of comparable alterations in AD and MCI points to the increased sleep latency and reduced SWS duration as the most reliable signs of disrupted sleep macrostructure in these clinical populations.

### NREM sleep

The main NREM sleep feature in the AD group compared to HC is represented by the significant reduction of the EEG activity in the sigma band at the temporo-posterior areas. Given the overlap between sigma band and sleep spindles frequency range (De Gennaro and Ferrara, 2003), our finding mirrors the previously described reduction of parietal spindle density in AD/MCI (Gorgoni et al., 2016). Sleep spindles are generated through the interaction between thalamic reticular nucleus and corticothalamic networks (De Gennaro and Ferrara, 2003), with a hippocampal involvement (Andrade et al., 2011). Accordingly, the reduction of sigma activity may represent an EEG expression of the thalamic and hippocampal damages, and the loss of cortical connectivity with such subcortical structures that characterize AD and MCI (de Jong et al., 2008; Yi et al., 2016). It is worth noting that the reduced sigma power at the temporal areas discriminates between MCI and HC, and not between MCI and AD, while the occipital sigma decrease is significantly different between AD and MCI. This regional specificity could be interpreted as a sign of the topological targeting and progression of the neurodegeneration process that is the earlier involvement of the temporal areas than the occipital ones in the neurodegeneration processes underlying the AD pathology (Braak and Braak, 1997).

No significant between-group differences have been found in the delta activity of NREM sleep. Considering the hypothesis of slow wave activity (SWA) impairment as a concurring factor in AD development (Cordone et al., 2019; Lim et al., 2014), this result may appear surprising. However, a significant reduction of the frontal K-complexes density during NREM sleep has been recently found in AD compared to elderly controls (De Gennaro et al., 2017) and MCI (Reda et al., 2017), with no differences in the ≤1Hz SWA. Given that the K-complexes are EEG ‘transients’ characterized by high amplitude and low frequency (≤1Hz), such dissociation between K-complexes and ≤1Hz SWA power suggests that the assessment of EEG power could not be sensitive enough to discriminate between these different coexistent phenomena. Future studies should further investigate SWA alterations during NREM sleep in AD, focusing on the analysis of the different contributions of K-complexes, slow-oscillations (≤1Hz), and higher-frequency SWA.

### REM sleep

Our results clearly show that, during REM sleep, the EEG slowing is the main phenomenon differentiating the three groups. In detail, the increase of the delta power on temporo-frontal regions and the reduction in the beta band on the temporal regions discriminate both the AD and MCI groups from the HC. The main differences between the AD and MCI groups involve the decrease of alpha, sigma, and beta activities at the occipital areas in the former instead. Such a result may reflect, once again, the later involvement of the occipital regions in the process of progressive neurodegeneration. Thus, the increase of cortical activity at the lowest frequencies characterizes the REM sleep EEG as early as in the MCI status. On the other hand, the EEG slowing component represented by the posterior reduction of the high-frequency activity appears as more specific feature of the AD condition.

This EEG pattern confirms the presence of strong and widespread alterations in EEG activity during REM sleep in AD and MCI, encompassing the whole spectrum with frequency-specific topography and representing a very sensitive index of the disease progression (Brayet et al., 2016; Hassainia et al., 1997; Montplaisir et al., 1996, 1998; Petit et al., 1992, 1993). The reason is likely linked to the crucial and exclusive role played by the basal forebrain cholinergic system in maintaining cortical arousal during REM sleep (Hobson et al., 1975; Sakai et al., 1990), and by the early impairment of this system in the course of the disease (Whitehouse et al., 1982). Moreover, our results confirm that the spectral profile of REM sleep could represent a better EEG marker of AD than that of the awake condition, at least in terms of index of the basal cholinergic activity (Montplaisir et al., 1998).

### Waking EEG before and after sleep

As expected, the waking EEG in the AD population is characterized by a significant increase of the delta activity with a frontotemporal topography in the evening and the concurrent involvement of the medial and posterior regions in the morning. The waking EEG in the evening also revealed a significant reduction of the occipital alpha power, not visible in the morning EEG. A similar pattern characterizes the EEG of the MCI. The prefrontal and midline frontal delta power in the morning significantly differed in all three groups, with AD showing the highest values, followed by MCI. The delta power at the central, parietal, and temporal regions instead did not differ between MCI and HC but only between AD and MCI.

Thus, our results substantially confirm the increase of slow-frequency cortical activity at frontotemporal areas as the main feature of the waking EEG in AD and MCI, supporting one of the most consolidated findings in the field (Babiloni et al, 2006, 2013, 2015, 2016; Jeong, 2004). They also prompt the importance of the time of day of the recording. Indeed, in our sample, the cortical activity in the alpha band showed significant between-group differences only in the evening, with a significant reduction affecting the two patients' groups in a similar manner. Instead, the delta activity showed significant differences in both the evening and the morning recordings. However, only in the morning, besides the AD *versus* HC comparison, both the AD *versus* MCI and the MCI *versus* HC comparisons reveal a significant and progressive delta power increase that gets worse with the severity of the disease. In the evening, the homeostatic sleep pressure modulates delta activity and likely acts as a confounding factor, explaining the different pattern of results compared to the morning.

By deepening the investigation on the influence of homeostatic factors, the present study highlights a new phenomenon characterizing the AD and MCI groups: the gradual disappearance of the overnight changes in the delta power with the worsening of the disease. In healthy conditions, slow-frequency EEG activity is lowest in the morning, progressively higher with the time spent awake during the day (Cacot et al., 1995; Finelli et al., 2000; Gorgoni et al., 2014) and returns to baseline levels after a night of sleep (Cacot et al., 1995; Corsi-Cabrera et al., 1992; Cummings et al., 2001). It is considered a reliable EEG index of sleep pressure (De Gennaro et al., 2007; Finelli et al., 2000; Gorgoni et al., 2014), that is the need to sleep in order to restore cortical processes saturated during wake. Accordingly, the changes in resting EEG after sleep could be considered an index of the efficiency of the sleep-dependent restoring processes (Corsi-Cabrera et al., 1992). In this view, our results suggest that sleep in AD, and to a lesser extent in MCI, does not thoroughly fulfill this compensative function. The significant and mostly exclusive correlation in the AD group among the overnight changes in waking delta activity and the reduction in high-frequency EEG activity during NREM and REM sleep suggest that the sleep alterations are strictly associated to the lack of morning vs. evening EEG changes.

Interestingly, the circadian variations of β-amyloid levels in the cerebrospinal fluid (CSF) show a similar reduction in the presence of β-amyloid aggregation (Boespflug and Iliff, 2018). In healthy individuals, CSF β-amyloid levels show wake-dependent increases and a sleep-dependent decline, while these dynamics are impaired in the AD models (Igarashi et al., 2014; Lucey et al., 2017; Suzuki et al., 2015). Further studies should investigate on the possible link between these two phenomena.

### The EEG slowing during wakefulness and REM sleep

The evaluation of the synthetic index of EEG slowing indicates a higher sensitivity of REM sleep compared to wakefulness in revealing significant differences among the three groups and the temporo-occipital regions as the regional marker of this phenomenon. Furthermore, there is a strong linear correlation in AD between the EEG slowing in these areas during REM sleep and the EEG slowing during wakefulness, which is clearly traceable in the MCI condition and much weaker in healthy elderly.

The MCI group also shows a peculiar influence of the homeostatic pressure on the correlation levels, almost absent in the other two groups. In the evening, when the sleep pressure is high, the correlation level in MCI was halfway along a continuum from HC to AD. In the morning, when the homeostatic sleep pressure should be minimal, the correlation increased, moving closer to the level shown by AD. This finding could be linked to some degree of preservation of the homeostatic process in the MCI group, as revealed by the comparison of the resting EEG before and after sleep. In particular, two distinct sources could influence the EEG slowing index to the a different extent in the three groups: a component directly associated to the neurodegenerative process in cortical and subcortical structures (Dringenberg, 2000), affecting both low and high frequency activity in opposite directions (henceforth the neurodegenerative component), and a component linked to the homeostatic sleep pressure (henceforth the homeostatic component), mainly driving the EEG slow-frequency activity during wakefulness in healthy condition (De Gennaro et al., 2007; Finelli et al., 2000; Gorgoni et al., 2014). The homeostatic component would be prominent in determining the EEG slowing during wake in HC. The two components would contribute to the EEG slowing during wakefulness in the MCI group. Conversely, the neurodegenerative component would be the critical source in the AD, due to the more severe degree of loss of functionality of the cholinergic system. The EEG slowing during REM sleep in posterior regions could be considered an index more likely reflecting the neurodegenerative process of the cholinergic system (Montplaisir et al., 1998) rather than reflecting homeostatic factors. Accordingly, its correlation with the EEG slowing during wakefulness is weak in the HC group, since it mainly depends on the homeostatic component in both the evening and the morning, and maximal in AD. In the MCI, the correlation increases from evening to morning, because of the sleep-related reduction of the homeostatic component contribution that allows the neurodegenerative component of the EEG slowing to emerge. In this view, our findings support the possibility of adopting the EEG slowing during REM sleep also as a representative index of the neurodegenerative component of the EEG slowing during wakefulness, excluding the confounding bias of homeostatic factors.

### Relationship between EEG alterations and cognitive impairment

The EEG index that showed the strongest correlation with cognitive deterioration is the synthetic index of the EEG slowing during REM sleep. This result confirms previous findings (Montplaisir et al., 1996), and it also suggests that this composite index may be better suited as a disease marker than others based on cortical activity in a single frequency band measured during REM and NREM sleep or resting state, as well as than the same index evaluated during wakefulness.

### Conclusions

The current study provides a detailed picture of the topographic and frequency-specific alterations in the EEG of AD and MCI populations during sleep and wakefulness compared with healthy elderly, highlighting their relationships and assessing them in the largest well-controlled cohort studied so far.

Beyond the difficulty of falling asleep and the reduction in SWS, we found that the sleep architecture is quite preserved in our AD and MCI samples. In this sense, the EEG slowing in these populations should not be seen as a direct secondary consequence of poor sleep. However, the sleep onset delay and the SWS impairment support an involvement of the sleep-wake control system in the neurodegenerative process. Along this line, the original finding of the gradual disappearance of the across-night changes in waking EEG activity in MCI and AD, whose reduction follows the severity of the disease, points to a progressive loss of the restorative function of sleep on diurnal EEG activity. However, this phenomenon should not strictly depend on increased intra-sleep wakefulness or reduced total sleep time, given the quite preserved sleep quality of the clinical samples in the current study.

Finally, we found a strong linear correlation between the EEG slowing during REM sleep and during wakefulness in the AD and MCI, suggesting that the two phenomena may share the same neuropathological mechanisms. Our findings also indicate that REM sleep could be a more sensible marker of the neurodegenerative process since early stages than the waking state, given that it is less affected by the possible confounding of homeostatic factors and the EEG slowing during REM sleep is the EEG index most correlated to the cognitive decline.

### Limitations of the study

Actually, one of the major limits of the present study is the lack of PET + radioligand or CSF beta-amyloid evaluation neither in AD nor in MCI. Moreover, the lack of appropriate clinical follow-up and/or the use of biomarkers for MCI-prodromal-to-dementia diagnosis did not allow us to discriminate the percentage of MCI already affected from those who will never be demented, suggesting to look with caution at the findings in MCI. Meanwhile, the MCI condition, as approached here, faithfully reflects the MCI diagnosis and management in the real world. Future trials should address these crucial points.

### Resource availability

#### Lead contact

Further information and requests for resources should be directed to and will be fulfilled by the lead contact, Luigi De Gennaro (luigi.degennaro@uniroma1.it).

#### Materials availability

This study did not generate new unique reagents.

#### Data and code availability

The datasets supporting the current study have not been deposited in a public repository but are available from the corresponding author on request.

## Methods

All methods can be found in the accompanying transparent methods supplemental file.

## Supplemental information

Document S1. Transparent methods, Figures S1 and S2, and Tables S1–S7

## References

[bib1] Andrade K.C., Spoormaker V.I., Dresler M., Wehrle R., Holsboer F., Samann P.G., Czisch M. (2011). Sleep spindles and hippocampal functional connectivity in human NREM sleep. J. Neurosci..

[bib2] Babiloni C., Binetti G., Cassetta E., Forno G.D., Percio C. Del, Ferreri F., Ferri R., Frisoni G., Hirata K., Lanuzza B. (2006). Sources of cortical rhythms change as a function of cognitive impairment in pathological aging: a multicenter study. Clin. Neurophysiol..

[bib3] Babiloni C., Lizio R., Del Percio C., Marzano N., Soricelli A., Salvatore E., Ferri R., Cosentino F.I.I., Tedeschi G., Montella P. (2013). Cortical sources of resting state EEG rhythms are sensitive to the progression of early stage Alzheimer’s disease. J. Alzheimer’s Dis..

[bib4] Babiloni C., Lizio R., Marzano N., Capotosto P., Soricelli A., Triggiani A.I., Cordone S., Gesualdo L., Del Percio C. (2015). Brain neural synchronization and functional coupling in Alzheimer’s disease as revealed by resting state EEG rhythms. Int. J. Psychophysiol..

[bib5] Babiloni C., Triggiani A.I., Lizio R., Cordone S., Tattoli G., Bevilacqua V., Soricelli A., Ferri R., Nobili F., Gesualdo L. (2016). Classification of single normal and Alzheimer’s disease individuals from cortical sources of resting state EEG rhythms. Front. Neurosci..

[bib6] Boespflug E.L., Iliff J.J. (2018). The emerging relationship between interstitial fluid–cerebrospinal fluid exchange, amyloid-β, and sleep. Biol. Psychiatry.

[bib7] Braak H., Braak E. (1997). Frequency of stages of alzheimer-related lesions in different age categories. Neurobiol. Aging.

[bib8] Brayet P., Petit D., Frauscher B., Gagnon J.-F., Gosselin N., Gagnon K., Rouleau I., Montplaisir J. (2016). Quantitative EEG of rapid-eye-movement sleep: a marker of amnestic mild cognitive impairment. Clin. EEG Neurosci..

[bib9] Cacot P., Tesolin B., Sebban C. (1995). Diurnal variations of EEG power in healthy adults. Electroencephalogr. Clin. Neurophysiol..

[bib10] Claus J.J., Ongerboer de Visser B.W., Bour L.J., Walstra G.J.M., Hijdra A., Verbeeten B., van Royen E.A., Kwa V.I.H., van Gool W.A. (2000). Determinants of quantitative spectral electroencephalography in early Alzheimer’s disease: cognitive function, regional cerebral blood flow, and computed tomography. Dement. Geriatr. Cogn. Disord..

[bib11] Cordone S., Annarumma L., Rossini P.M., De Gennaro L. (2019). Sleep and β-amyloid deposition in Alzheimer disease: insights on mechanisms and possible innovative treatments. Front. Pharmacol..

[bib12] Corsi-Cabrera M., Ramos J., Arce C., Guevara M.A., Ponce-de León M., Lorenzo I. (1992). Changes in the waking EEG as a consequence of sleep and sleep deprivation. Sleep.

[bib13] Cummings L., Dane A., Rhodes J., Lynch P., Hughes A.M. (2001). Diurnal variation in the quantitative EEG in healthy adult volunteers. Br. J. Clin. Pharmacol..

[bib14] Curcio G., Ferrara M., Pellicciari M.C., Cristiani R., De Gennaro L. (2003). Effect of total sleep deprivation on the landmarks of stage 2 sleep. Clin. Neurophysiol..

[bib15] D’Rozario A.L., Chapman J.L., Phillips C.L., Palmer J.R., Hoyos C.M., Mowszowski L., Duffy S.L., Marshall N.S., Benca R., Mander B. (2020). Objective measurement of sleep in mild cognitive impairment: a systematic review and meta-analysis. Sleep Med. Rev..

[bib16] De Gennaro L., Ferrara M. (2003). Sleep spindles : an overview. Sleep Med..

[bib17] De Gennaro L., Gorgoni M., Reda F., Lauri G., Truglia I., Cordone S., Scarpelli S., Mangiaruga A., D’atri A., Lacidogna G. (2017). The fall of sleep K-complex in alzheimer disease. Sci. Rep..

[bib18] De Gennaro L., Marzano C., Veniero D., Moroni F., Fratello F., Curcio G., Ferrara M., Ferlazzo F., Novelli L., Concetta Pellicciari M. (2007). Neurophysiological correlates of sleepiness: a combined TMS and EEG study. Neuroimage.

[bib19] de Jong L.W., van der Hiele K., Veer I.M., Houwing J.J., Westendorp R.G.J., Bollen E.L.E.M., de Bruin P.W., Middelkoop H.A.M., van Buchem M.A., van der Grond J. (2008). Strongly reduced volumes of putamen and thalamus in Alzheimer’s disease: an MRI study. Brain.

[bib20] Dringenberg H.C. (2000). Alzheimer’s disease: more than a ‘cholinergic disorder’’ — evidence that cholinergic–monoaminergic interactions contribute to EEG slowing and dementia. Behav. Brain Res..

[bib21] Finelli L.A., Baumann H., Borbély A.A., Achermann P. (2000). Dual electroencephalogram markers of human sleep homeostasis: correlation between theta activity in waking and slow-wave activity in sleep. Neuroscience.

[bib22] Galluzzi S., Cimaschi L., Ferrucci L., Frisoni G.B. (2001). Mild cognitive impairment: clinical features and review of screening instruments. Aging Clin. Exp. Res..

[bib23] Gorgoni M., Ferlazzo F., Ferrara M., Moroni F., D’Atri A., Fanelli S., Gizzi Torriglia I., Lauri G., Marzano C., Rossini P.M., De Gennaro L. (2014). Topographic electroencephalogram changes associated with psychomotor vigilance task performance after sleep deprivation. Sleep Med..

[bib24] Gorgoni M., Lauri G., Truglia I., Cordone S., Sarasso S., Scarpelli S., Mangiaruga A., D’Atri A., Tempesta D., Ferrara M. (2016). Parietal fast sleep spindle density decrease in Alzheimer’s disease and amnesic mild cognitive impairment. Neural Plast..

[bib25] Hassainia F., Petit D., Nielsen T., Gauthier S., Montplaisir J. (1997). Quantitative EEG and statistical mapping of wakefulness and REM sleep in the evaluation of mild to moderate Alzheimer’s disease. Eur. Neurol..

[bib26] Hobson J., McCarley R., Wyzinski P. (1975). Sleep cycle oscillation: reciprocal discharge by two brainstem neuronal groups. Science.

[bib27] Holth J.K., Fritschi S.K., Wang C., Pedersen N.P., Cirrito J.R., Mahan T.E., Finn M.B., Manis M., Geerling J.C., Fuller P.M. (2019). The sleep-wake cycle regulates brain interstitial fluid tau in mice and CSF tau in humans. Science.

[bib28] Holth J.K., Mahan T.E., Robinson G.O., Rocha A., Holtzman D.M. (2017). Altered sleep and EEG power in the P301S Tau transgenic mouse model. Ann. Clin. Transl. Neurol..

[bib29] Igarashi H., Suzuki Y., Kwee I.L., Nakada T. (2014). Water influx into cerebrospinal fluid is significantly reduced in senile plaque bearing transgenic mice, supporting beta-amyloid clearance hypothesis of Alzheimer’s disease. Neurol. Res..

[bib30] Jelic V., Shigeta M., Julin P., Almkvist O., Winblad B., Wahlund L.-O. (1996). Quantitative electroencephalography power and coherence in Alzheimer’s disease and mild cognitive impairment. Dement. Geriatr. Cogn. Disord..

[bib31] Jeong J. (2004). EEG dynamics in patients with Alzheimer’s disease. Clin. Neurophysiol..

[bib32] Ju Y.-E.S., Ooms S.J., Sutphen C., Macauley S.L., Zangrilli M.A., Jerome G., Fagan A.M., Mignot E., Zempel J.M., Claassen J.A.H.R., Holtzman D.M. (2017). Slow wave sleep disruption increases cerebrospinal fluid amyloid-β levels. Brain.

[bib33] Kang J.-E., Lim M.M., Bateman R.J., Lee J.J., Smyth L.P., Cirrito J.R., Fujiki N., Nishino S., Holtzman D.M. (2009). Amyloid- dynamics are regulated by orexin and the sleep-wake cycle. Science.

[bib34] Klinzing J.G., Niethard N., Born J. (2019). Mechanisms of systems memory consolidation during sleep. Nat. Neurosci..

[bib35] Lim A.S.P., Yu L., Kowgier M., Schneider J.A., Buchman A.S., Bennett D.A. (2013). Modification of the relationship of the apolipoprotein E ε4 allele to the risk of alzheimer disease and neurofibrillary tangle density by sleep. JAMA Neurol..

[bib36] Lim M.M., Gerstner J.R., Holtzman D.M. (2014). The sleep-wake cycle and Alzheimer’s disease: what do we know?. Neurodegener. Dis. Manag..

[bib37] Lucey B.P., Hicks T.J., McLeland J.S., Toedebusch C.D., Boyd J., Elbert D.L., Patterson B.W., Baty J., Morris J.C., Ovod V. (2018). Effect of sleep on overnight cerebrospinal fluid amyloid β kinetics. Ann. Neurol..

[bib38] Lucey B.P., Mawuenyega K.G., Patterson B.W., Elbert D.L., Ovod V., Kasten T., Morris J.C., Bateman R.J. (2017). Associations between β-amyloid kinetics and the β-amyloid diurnal pattern in the central nervous system. JAMA Neurol..

[bib39] Lucey B.P., McCullough A., Landsness E.C., Toedebusch C.D., McLeland J.S., Zaza A.M., Fagan A.M., McCue L., Xiong C., Morris J.C. (2019). Reduced non–rapid eye movement sleep is associated with tau pathology in early Alzheimer’s disease. Sci. Transl. Med..

[bib40] Mander B.A., Marks S.M., Vogel J.W., Rao V., Lu B., Saletin J.M., Ancoli-Israel S., Jagust W.J., Walker M.P. (2015). β-amyloid disrupts human NREM slow waves and related hippocampus-dependent memory consolidation. Nat. Neurosci..

[bib41] Marzano C., Ferrara M., Curcio G., De Gennaro L. (2010). The effects of sleep deprivation in humans: topographical electroencephalogram changes in non-rapid eye movement (NREM) sleep versus REM sleep. J. Sleep Res..

[bib42] Menkes-Caspi N., Yamin H.G., Kellner V., Spires-Jones T.L., Cohen D., Stern E.A. (2015). Pathological tau disrupts ongoing network activity. Neuron.

[bib43] Montplaisir J., Petit D., Gauthier S., Gaudreau H., Décary A. (1998). Sleep disturbances and EEG slowing in Alzheimer’s disease. Sleep Res. Online.

[bib44] Montplaisir J., Petit D., McNamara D., Gauthier S. (1996). Comparisons between SPECT and quantitative EEG measures of cortical impairment in mild to moderate Alzheimer’s disease. Eur. Neurol..

[bib45] Ooms S., Overeem S., Besse K., Rikkert M.O., Verbeek M., Claassen J.A.H.R. (2014). Effect of 1 night of total sleep deprivation on cerebrospinal fluid β-amyloid 42 in healthy middle-aged men. JAMA Neurol..

[bib46] Peter-Derex L., Yammine P., Bastuji H., Croisile B. (2015). Sleep and Alzheimer’s disease. Sleep Med. Rev..

[bib47] Petersen R.C., Doody R., Kurz A., Mohs R.C., Morris J.C., Rabins P.V., Ritchie K., Rossor M., Thal L., Winblad B. (2001). Current concepts in mild cognitive impairment. Arch. Neurol..

[bib48] Petit D., Gagnon J.-F., Fantini M.L., Ferini-Strambi L., Montplaisir J. (2004). Sleep and quantitative EEG in neurodegenerative disorders. J. Psychosom. Res..

[bib49] Petit D., Lorrain D., Gauthier S., Montplaisir J. (1993). Regional spectral analysis of the REM sleep EEG in mild to moderate Alzheimer’s disease. Neurobiol. Aging.

[bib50] Petit D., Montplaisir J., Lorrain D., Gauthier S. (1992). Spectral analysis of the rapid eye movement sleep electroencephalogram in right and left temporal regions: a biological marker of Alzheimer’s disease. Ann. Neurol..

[bib51] Reda F., Gorgoni M., Lauri G., Truglia I., Cordone S., Scarpelli S., Mangiaruga A., D’Atri A., Ferrara M., Lacidogna G. (2017). In search of sleep biomarkers of Alzheimer’s disease: K-complexes do not discriminate between patients with mild cognitive impairment and healthy controls. Brain Sci..

[bib52] Sakai K., El Mansari M., Lin J.S., Zhang J.G., Vanni-Mercier G., Mancia M., Marini G. (1990). The posterior hypothalamus in the regulation of wakefulness and paradoxical sleep. The Diencephalon and Sleep.

[bib53] Scheltens P., Fox N., Barkhof F., De Carli C. (2002). Structural magnetic resonance imaging in the practical assessment of dementia: beyond exclusion. Lancet Neurol..

[bib54] Suzuki Y., Nakamura Y., Yamada K., Igarashi H., Kasuga K., Yokoyama Y., Ikeuchi T., Nishizawa M., Kwee I.L., Nakada T. (2015). Reduced CSF water influx in Alzheimer’s disease supporting the β-amyloid clearance hypothesis. PLoS One.

[bib55] Villa C., Ferini-Strambi L., Combi R. (2015). The synergistic relationship between Alzheimer’s disease and sleep disorders: an update. J. Alzheimer’s Dis..

[bib56] Whitehouse P., Price D., Struble R., Clark A., Coyle J., Delon M. (1982). Alzheimer’s disease and senile dementia: loss of neurons in the basal forebrain. Science.

[bib57] Winer J.R., Mander B.A., Helfrich R.F., Maass A., Harrison T.M., Baker S.L., Knight R.T., Jagust W.J., Walker M.P. (2019). Sleep as a potential biomarker of tau and β-amyloid burden in the human brain. J. Neurosci..

[bib58] Winer J.R., Mander B.A., Kumar S., Reed M., Baker S.L., Jagust W.J., Walker M.P. (2020). Sleep disturbance forecasts β-amyloid accumulation across subsequent years. Curr. Biol..

[bib59] Xie L., Kang H., Xu Q., Chen M.J., Liao Y., Thiyagarajan M., O’Donnell J., Christensen D.J., Nicholson C., Iliff J.J. (2013). Sleep drives metabolite clearance from the adult brain. Science.

[bib60] Yi H.-A., Möller C., Dieleman N., Bouwman F.H., Barkhof F., Scheltens P., van der Flier W.M., Vrenken H. (2016). Relation between subcortical grey matter atrophy and conversion from mild cognitive impairment to Alzheimer’s disease. J. Neurol. Neurosurg. Psychiatry.

