# Supplementary material for: EEG alterations during wake and sleep in mild cognitive impairment and Alzheimer's disease
Source: iScience. 2021 Apr 1;24(4):102386. doi: 10.1016/j.isci.2021.102386 (PMC8086022; doi:10.1016/j.isci.2021.102386)
Supplement: Document S1. Transparent methods, Figures S1 and S2, and Tables S1–S7 [file mmc1.pdf]

iScience, Volume *24*

Supplemental information

EEG alterations during wake and sleep in mild cognitive impairment and Alzheimer's disease

Aurora D'Atri, Serena Scarpelli, Maurizio Gorgoni, Ilaria Truglia, Giulia Lauri, Susanna Cordone, Michele Ferrara, Camillo Marra, Paolo Maria Rossini, and Luigi De Gennaro

Supplemental Data Items

Supplemental Figures

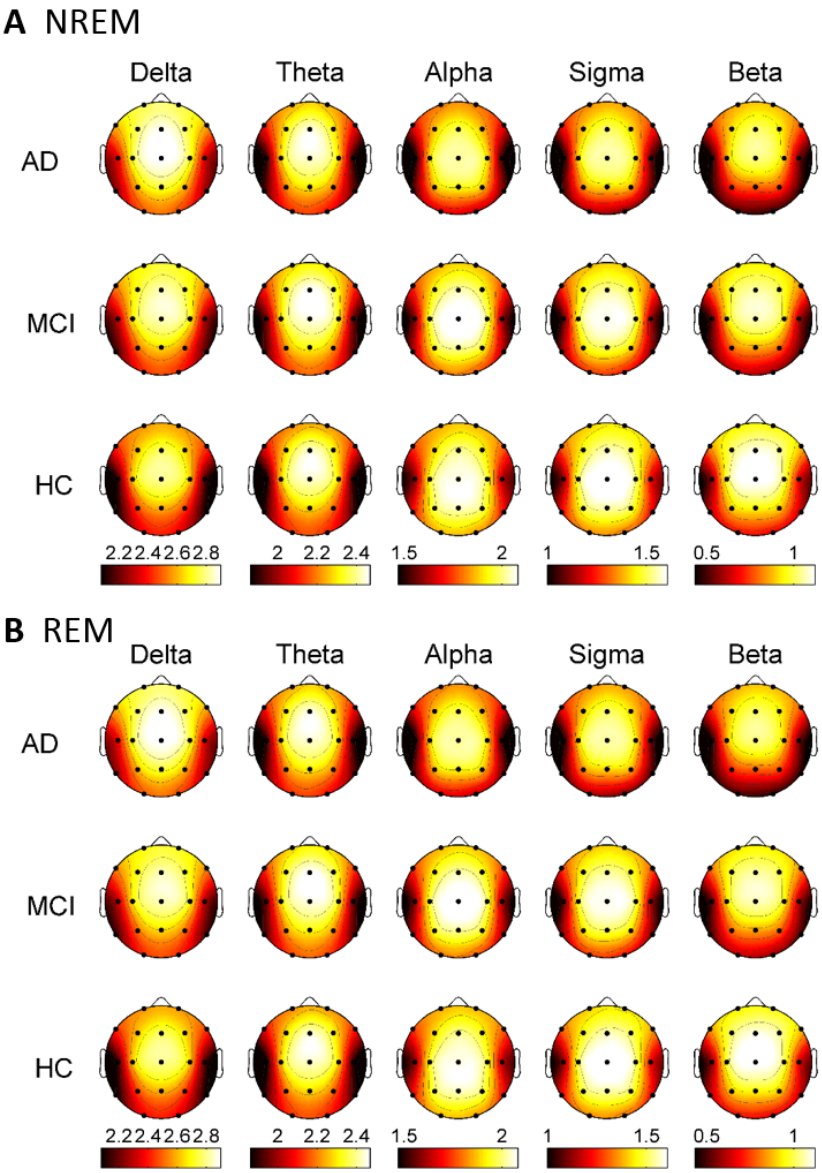

Figure S1. EEG spectral power during NREM and REM sleep in AD, MCI and HC groups, **Related to Figure 1**. Topographic maps of the mean spectral power (log-transformed) during NREM (panel A) and REM sleep (panel B) in AD (1st row), MCI (2nd row) and HC (3rd row). The topographic maps are scaled between minimal and maximal values of the three groups within each frequency band for each condition**.**

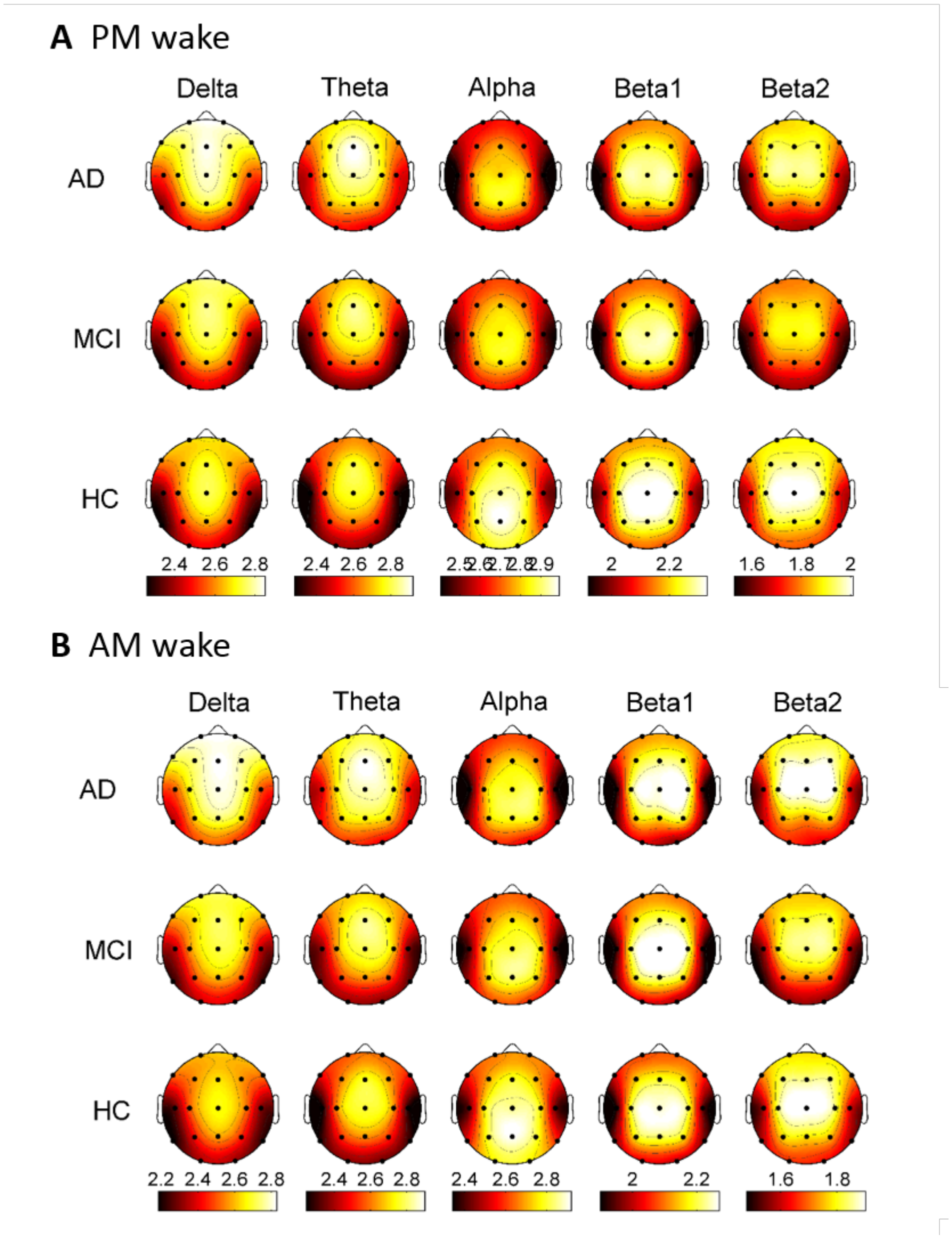

Fig. S2. EEG spectral power during evening and morning wakefulness in AD, MCI and HC groups, **Related to Figure 2.** Topographic maps of the mean spectral power (log-transformed) during evening (PM, panel A) and morning (AM) wake (panel B) in AD (1st row), MCI (2nd row) and HC (3rd row). The topographic maps are scaled between minimal and maximal values of the three groups within each frequency band.

Supplemental Tables

Table S1. Results of the significant one-way ANOVAs (AD *vs*. MCI *vs*. HC) on the EEG power of the NREM and REM sleep ($p \leq 0.0102$) and the corresponding *post-hoc* unpaired t-tests ($p \leq 0.05$). Related to Figure 1.

**Sleep stage**	**EEG site**	**Freq band**			**AD *vs*. HC**		**MCI *vs*. HC**		**AD *vs*. MCI**	
			$F_{2,147}$	p	t_{98}	p	t_{98}	p	t_{98}	p
**NREM**	**O1**	Sigma	7.87	0.00057	-3.75	0.0003	-	-	-2.20	0.0301
	**O2**	Sigma	9.39	0.00015	-4.11	0.00008	-2.00	0.048	-2.46	0.0156
	**P3**	Sigma	5.35	0.0058	-3.25	0.0016	-	-	2.36	0.020
	**Pz**	Sigma	5.54	0.0048	-3.28	0.0015	-	-	2.49	0.014
	**T3**	Alpha	5.83	0.0037	-3.21	0.0018	-	-	-1.99	0.049
	**T5**	Alpha	6.61	0.0018	-3.53	0.0064	-	-	-	-
		Sigma	7.82	0.00059	-3.97	0.00014	-2.11	0.038	-	-
	**T6**	Sigma	5.54	0.0048	-3.18	0.0003	-2.13	0.036	-	-
**REM**			$F_{2,141}$	p	t_{93}	p	t_{96}	p	t_{93}	p
	**Fp1**	Delta	8.12	0.00046	4.10	0.000089	2.85	0.0053	-	-
	**Fp2**	Delta	8.49	0.00033	4.18	0.000065	3.04	0.0030	-	-
	**F3**	Delta	4.96	0.0083	3.11	0.0025	2.02	0.046	-	-
	**F7**	Delta	7.12	0.0011	4.03	0.00012	2.35	0.021	-	-
	**F8**	Delta	5.32	0.0059	3.39	0.0010	2.22	0.029	-	-
	**O1**	Alpha	5.38	0.0056	-2.95	0.0041	-	-	-2.32	0.022
		Sigma	7.26	0.0010	-3.53	0.00065	-	-	-2.40	0.019
		Beta	6.64	0.0018	-3.41	0.00097	-	-	-2.36	0.021
	**O2**	Alpha	5.49	0.0051	-2.99	0.0035	-	-	-2.33	0.022
		Sigma	7.80	0.00061	-3.63	0.00047	-	-	-2.47	0.015
		Beta	6.81	0.0015	-3.42	0.00092	-	-	-2.11	0.037
	**T3**	Delta	9.79	0.00010	4.41	0.000028	3.27	0.0015	-	-
	**T4**	Delta	6.94	0.0013	3.83	0.00023	2.63	0.0099	3.28	0.0406
	**T5**	Delta	4.80	0.0096	3.16	0.0021	-	-	-	-
		Sigma	5.04	0.0077	-3.06	0.0029	-	-	-	-
		Beta	5.39	0.0055	-3.15	0.0022	-2.18	0.031	-	-
	**T6**	Delta	5.60	0.0045	3.52	0.00067	-	-	-	-
		Beta	5.04	0.0077	-2.83	0.0057	-2.36	0.020	-	-

Abbreviations: AD, Alzheimer's disease; MCI, mild cognitive impairment; HC, healthy control; NREM, non-REM.

Table S2. Results of the significant one-way ANOVAs (AD *vs*. MCI *vs*. HC) on the EEG power of the evening (PM) and morning (AM) wakefulness ($p \leq 0.0102$) and the corresponding *post-hoc* unpaired t-tests ($p \leq 0.05$). Related to Figure 2.

Wake time	EEG site	Freq band	$F_{2,141}$	p	AD *vs*. HC		MCI *vs*. HC		AD *vs*. MCI	
					t_{93}	p	t_{97}	p	t_{92}	P
**PM**	**Fp1**	Delta	5.06	0.0075	3.15	0.0022	-	-	-	-
	**Fp2**	Delta	5.65	0.0044	3.32	0.0013	-	-	-	-
	**F8**	Delta	5.10	0.0072	2.98	0.0037	-	-	2.14	0.035
	**O2**	Alpha	5.40	0.0055	-2.96	0.0040	-2.41	0.018	-	-
	**T4**	Delta	5.47	0.0051	2.95	0.0040	2.63	0.0099	2.52	0.013
**AM**			$F_{2,140}$	p	t_{92}	p	t_{97}	p	t_{91}	P
	**C3**	Delta	5.56	0.0048	3.03	0.0032	-	-	2.15	0.034
	**C4**	Delta	6.17	0.0027	3.25	0.0016	-	-	-	-
	**Cz**	Delta	4.74	0.010	2.71	0.0079	-	-	2.22	0.029
	**Fp1**	Delta	11.07	0.000034	4.41	0.000028	2.54	0.011	2.36	0.020
	**Fp2**	Delta	9.85	0.00010	4.15	0.000073	2.25	0.027	2.38	0.019
	**F3**	Delta	7.11	0.0011	3.50	0.00072	-	-	2.13	0.036
	**F4**	Delta	8.84	0.00024	3.95	0.00015	2.03	0.045	2.33	0.022
	**F7**	Delta	9.89	0.000096	4.03	0.00011	-	-	2.65	0.0096
	**F8**	Delta	9.87	0.000098	4.08	0.000095	-	-	2.84	0.0056
	**Fz**	Delta	7.81	0.00061	3.63	0.00046	2.10	0.038	2.09	0.040
	**P3**	Delta	6.19	0.0026	3.14	0.0023	-	-	2.36	0.020
	**P4**	Delta	5.92	0.0034	3.17	0.0021	-	-	2.35	0.021
	**Pz**	Delta	5.07	0.0075	2.75	0.0071	-	-	2.49	0.014
	**T3**	Delta	9.25	0.00017	4.01	0.00012	-	-	2.52	0.014
	**T4**	Delta	9.04	0.00020	3.92	0.00017	-	-	2.94	0.0042

Abbreviations: AD, Alzheimer's disease; MCI, mild cognitive impairment; HC, healthy control; PM wake, evening wake; AM wake, morning wake.

Table S3. Results of the significant interactions in the mixed-design ANOVAs *Group* (AD vs. MCI vs. HC) x *Time of day* (PM vs. AM) on the spectral power of the wake EEG ($p \leq 0.0102$) and the corresponding *post-hoc* paired t-tests (AM vs. PM) for each group ($p \leq 0.05$). Related to Figure 3 (Panel A, Panel B).

**EEG site**	**Frequency band**	$F_{2,139}$	***p***	**AD AM *vs.* PM**		**MCI AM *vs.* PM**		**HC AM *vs.* PM**	
				t_{42}	***p***	t_{48}	***p***	t_{49}	***P***
**C3**	Delta	5.00	0.0080	-	-	-2.67	0.010	-5.22	3.60×10^{-6}
**C4**	Delta	5.17	0.0068	-	-	-2.19	0.033	-5.02	7.24×10^{-6}
**F3**	Delta	5.12	0.0071	-	-	-2.79	0.0076	-5.58	0.000017
**F4**	Delta	5.90	0.0035	-	-	-2.59	0.013	-5.49	0.000035
**F7**	Delta	4.76	0.0010	-	-	-	-	-5.89	3.47×10^{-7}
**Fz**	Delta	5.14	0.0071	-	-	-2.56	0.014	-6.29	8.49×10^{-8}
**P3**	Delta	5.45	0.0053	-	-	-	-	-4.44	0.000050

Abbreviations: AD, Alzheimer's disease; MCI, mild cognitive impairment; HC, healthy control; PM wake, evening wake; AM wake, morning wake.

Table S4. Significant correlations (Pearson's *r*) between morning vs. evening changes in waking EEG delta power at F4 and the EEG power of the NREM and REM sleep in the AD group ($p \leq 0.0054$). No significant correlation has been found in MCI and HC groups. Related to Figure 3 (Panel C).

**EEG site**	**Sleep stage**	**Alpha**		**Sigma**		**Beta**	
		r	***p***	***r***	***p***	***r***	***p***
**C3**	NREM	-	-	-0.47	0.0027	-0.45	0.0040
**C4**	NREM	-	-	-0.52	0.00070	-0.54	0.00045
**Cz**	NREM	-	-	-0.47	0.0024	-0.46	0.0035
**F3**	NREM	-	-	-0.46	0.0029	-0.44	0.0047
**F4**	NREM	-	-	-0.49	0.0016	-0.44	0.0048
**F8**	NREM	-	-	-0.45	0.0042	-0.45	0.0042
**Fz**	NREM	-	-	-0.50	0.0013	-0.48	0.0022
**O1**	NREM	-0.50	0.0013	-0.53	0.00054	-0.59	0.000067
**O1**	REM	-0.49	0.0018	-	-	-0.52	0.00063
**O2**	NREM	-0.49	0.0014	-0.53	0.00053	-0.56	0.00021
**O2**	REM	-0.44	0.0048	-	-	-0.47	0.0025
**P3**	NREM	-0.48	0.0022	-0.52	0.00072	-0.54	0.00041
**P4**	NREM	-0.46	0.0033	-0.52	0.00061	-0.57	0.00013
**Pz**	NREM	-0.45	0.0040	-0.50	0.0011	-0.56	0.00021
**T5**	NREM	-0.50	0.0010	-0.57	0.00018	-0.54	0.00040
**T5**	REM	-0.45	0.0039	-0.47	0.0027	-0.51	0.00096
**T6**	NREM	-0.48	0.0020	-0.58	0.00012	-0.57	0.00014
**T6**	REM	-0.44	0.0050	-0.47	0.0028	-0.50	0.0012

Abbreviations: AD, Alzheimer's disease; MCI, mild cognitive impairment; HC, healthy control; NREM, non-REM.

Table S5. Results of the significant one-way ANOVAs (AD *vs*. MCI *vs*. HC) on the EEG slowing index of the morning and evening wakefulness, and the REM sleep ($p \leq 0.0102$), and the corresponding *post-hoc* unpaired t-tests ($p \leq 0.05$). Related to Figure 4.

EEG site	time of day/ sleep stage	$F_{2,133}$	p	AD *vs.* HC		MCI *vs.* HC		AD *vs.* MCI	
				t_{86}	p	t_{95}	p	t_{85}	p
**Fp1**	PM wake	5.33	0.0059	3.17	0.0021	2.44	0.017	-	-
**Fp2**	PM wake	5.19	0.0068	3.04	0.0032	2.60	0.011	-	-
**F7**	PM wake	5.31	0.0060	3.49	0.00077	2.08	0.04	-	-
	AM wake	4.89	0.0090	3.09	0.0027	2.03	0.045	-	-
**F8**	PM wake	5.16	0.0069	3.30	0.0014	2.27	0.025	-	-
**O1**	PM wake	6.03	0.0031	3.45	0.00086	1.99	0.050	-	-
	REM sleep	11.10	0.000035	4.26	0.000052	2.17	0.033	2.74	0.0074
	AM wake	5.03	0.0078	2.99	0.0036	1.99	0.050	-	-
**O2**	PM wake	6.89	0.0014	3.64	0.00047	2.33	0.022	-	-
	REM sleep	9.60	0.00013	3.90	0.000019	2.33	0.022	2.44	0.017
	AM wake	5.38	0.0057	3.07	0.0029	2.25	0.027	-	-
**P3**	PM wake	6.66	0.0017	3.64	0.00046	2.22	0.029	-	-
	REM sleep	6.09	0.0029	3.16	0.0022	-	-	-	-
	AM wake	4.97	0.0083	3.02	0.0033	-	-	-	-
**P4**	PM wake	5.36	0.0058	3.35	0.0012	2.20	0.030	-	-
**Pz**	PM wake	6.59	0.0019	3.59	0.00055	2.05	0.043	-	-
	REM sleep	6.21	0.0027	3.16	0.0022	-	-	2.00	0.048
**T3**	PM wake	8.90	0.00024	4.58	0.000015	2.80	0.0062	-	-
	REM sleep	6.29	0.0024	3.51	0.00071	2.26	0.026	-	-
	AM wake	6.91	0.0014	3.74	0.00033	2.27	0.025	-	-
**T4**	PM wake	7.34	0.00095	4.10	0.000094	2.21	0.030	-	-
	REM sleep	6.50	0.0020	3.42	0.00095	2.96	0.0039	-	-
	AM wake	5.69	0.0042	3.33	0.0013	1.99	0.050	-	-
**T5**	PM wake	8.69	0.00028	4.22	0.000061	2.68	0.0088	-	-
	REM sleep	9.54	0.00013	4.06	0.00011	2.67	0.0089	2.08	0.041
	AM wake	6.85	0.0015	3.57	0.00059	2.79	0.0064	-	-
**T6**	PM wake	9.25	0.00017	4.28	0.000048	3.11	0.0025	-	-
	REM sleep	6.98	0.0013	3.46	0.00084	2.81	0.0060	-	-

Abbreviations: AD, Alzheimer's disease; MCI, mild cognitive impairment; HC, healthy control; PM wake, evening wake; AM wake, morning wake

Table S6. Significant correlations (Pearson's *r*) between EEG slowing index during REM and the EEG slowing index during evening (PM) and morning (AM) wakefulness ($p \leq 0.0054$). Related to Figure 5.

EEG site	AD				MCI				HC			
	PM wakefulness		AM wakefulness		PM wakefulness		AM wakefulness		PM wakefulness		AM wakefulness	
	r	*p*	*r*	*p*	*r*	*p*	*r*	*p*	*r*	*p*	*r*	*p*
**C3**	0.70	5.83×10^{-7}	0.65	9.10×10^{-6}	0.47	0.00070	0.63	1.92×10^{-6}	-	0.020	-	-
**C4**	0.65	8.24×10^{-6}	0.65	9.27×10^{-6}	0.48	0.00055	0.60	6.10×10^{-6}	0.41	0.0033	-	-
**Cz**	0.71	3.55×10^{-7}	0.67	3.87×10^{-6}	0.51	0.00020	0.62	2.56×10^{-6}	0.45	0.0013	0.44	0.0016
**Fp1**	0.62	0.000028	0.58	0.00012	0.49	0.00039	0.62	2.46×10^{-6}	-	-	-	-
**Fp2**	0.54	0.00042	0.59	0.000067	0.49	0.00035	0.58	0.000013	-	-	-	-
**F3**	0.62	0.000030	0.53	0.00048	0.50	0.00026	0.64	1.07×10^{-6}	-	-	-	-
**F4**	0.60	0.000053	0.61	0.000033	0.49	0.00037	0.60	6.04×10^{-6}	-	-	-	-
**F7**	0.53	0.00049	0.45	0.0042	0.46	0.00104	0.54	0.000080	-	-	-	-
**F8**	-	-	0.47	0.0024	0.44	0.00189	0.52	0.00013	-	-	0.41	0.0035
**Fz**	0.70	5.76×10^{-7}	0.64	0.000012	0.50	0.00027	0.62	2.85×10^{-6}	0.39	0.0051	0.40	0.0047
**O1**	0.49	0.0016	0.46	0.0035	0.46	0.0010	0.58	0.000013	-	-	-	-
**O2**	0.47	0.0024	-	-	0.48	0.00061	0.56	0.000029	-	-	-	-
**P3**	0.70	7.17×10^{-7}	0.66	5.34×10^{-6}	0.54	0.000070	0.64	8.32×10^{-7}	-	-	-	-
**P4**	0.64	0.000012	0.67	3.81×10^{-6}	0.47	0.00082	0.58	0.000015	0.39	0.0050	-	-
**Pz**	0.70	6.94×10^{-7}	0.64	0.000010	0.50	0.00031	0.60	6.44×10^{-6}	0.44	0.0014	-	-
**T3**	-	-	-	-	0.48	0.00059	0.58	0.000019	-	-	-	-
**T4**	-	-	-	-	0.42	0.0032	0.49	0.00047	-	-	-	-
**T5**	0.50	0.0011	0.49	0.0017	0.48	0.00048	0.53	0.000098	-	-	-	-
**T6**	-	-	0.46	0.0029	0.44	0.0016	0.44	0.0018	-	-	0.45	0.0012

Table S7. Significant correlations (Pearson's *r*) between MMSE score and sigma power during NREM sleep, EEG slowing index during REM sleep, and EEG slowing index during evening (PM) and morning (AM) wakefulness ($p \leq 0.0054$). Related to Figure 6.

EEG site	Sigma power NREM sleep		EEG slowing PM wake		EEG slowing REM sleep		EEG slowing AM wake	
	r	*p*	*r*	*p*	*r*	*p*	*r*	*p*
**C3**	0.28	0.00054	-0.25	0.0028	-0.42	1.68×10^{-7}	-0.26	0.0017
**C4**	0.29	0.0003	-0.23	0.0054	-0.42	1.33×10^{-7}	-0.24	0.0039
**Cz**	0.29	0.00026	-0.24	0.0031	-0.39	1.05×10^{-6}	-0.24	0.0034
**Fp1**	-	-	-0.26	0.0018	-0.41	3.94×10^{-7}	-0.29	0.00039
**Fp2**	-	-	-	-	-0.42	1.58×10^{-7}	-0.28	0.00066
**F3**	0.25	0.0018	-	-	-0.42	2.30×10^{-7}	-0.27	0.00099
**F4**	0.25	0.0021	-0.23	0.0048	-0.41	2.84×10^{-7}	-0.27	0.0012
**F7**	0.27	0.0009	-0.29	0.00050	-0.46	5.88×10^{-9}	-0.30	0.00032
**F8**	0.29	0.00034	-0.23	0.0048	-0.45	1.03×10^{-8}	-0.29	0.00043
**Fz**	0.25	0.0019	-0.25	0.0022	-0.39	1.15×10^{-6}	-0.27	0.00090
**O1**	0.32	0.000077	-0.29	0.00037	-0.52	3.51×10^{-11}	-0.29	0.00052
**O2**	0.32	0.000055	-0.28	0.00065	-0.49	6.45×10^{-10}	-0.27	0.0010
**P3**	0.28	0.0006	-0.32	0.000098	-0.48	1.47×10^{-9}	-0.34	0.000043
**P4**	0.27	0.00067	-0.28	0.00055	-0.44	4.67×10^{-8}	-0.28	0.00086
**Pz**	0.31	0.000093	-0.31	0.00018	-0.45	1.75×10^{-8}	-0.32	0.000080
**T3**	0.24	0.0027	-0.32	0.00010	-0.50	2.80×10^{-10}	-0.33	0.000062
**T4**	0.26	0.0013	-0.27	0.0011	-0.48	1.68×10^{-9}	-0.31	0.00018
**T5**	0.31	0.00014	-0.34	0.000023	-0.53	8.00×10^{-12}	-0.29	0.00052
**T6**	0.29	0.00033	-0.30	0.00021	-0.49	3.80×10^{-10}	-	-

Abbreviations: AD, Alzheimer's disease; MCI, mild cognitive impairment; HC, healthy control; PM wake, evening wake; AM wake, morning wake; NREM, non-REM.

Transparent Methods

Patients and healthy controls

Fifty consecutive subjects newly diagnosed Alzheimer's Disease (20 males; age range: 55–85 years; mean: 73 ± 6.9) and fifty subjects with a diagnosis of mild cognitive impairment (25 males; age range: 55–84 years; mean: 72 ± 6.8) from the Neurology Department of Foundation Policlinico Universitario A. Gemelli – IRCCS were enrolled for the study. The diagnostic procedure involved structured clinical evaluation, brain neuroimaging (MRI or CT), and a battery of neuropsychological tests assessing their functionality in different cognitive domains (attention, executive and visuo-spatial functions, memory, language) and their functional status (Activities of Daily Living/Instrumental Activities of Daily Living questionnaire). In particular, memory assessment included Rey's Auditory Verbal Learning (RAVLT, (Carlesimo et al., 1996), involving immediate/delayed recall and delayed recognition, delayed recall of the Rey figure (Rey, 1983), delayed recall of a three word list (Chandler et al., 2004), delayed recall of a story (Spinnler et al., 1987), memory span for numbers and visuo-spatial memory span. MRI data were available for a small group of AD and MCI participants only and were not included in the study.

AD diagnosis conformed to the National Institute on Aging-Alzheimer's Association workgroups (McKhann et al., 2011) and DSM-IV criteria. According to the guidelines and clinical standards for MCI diagnosis (Petersen et al., 2001; Portet, 2006; Zaudig, 1992), the MCI sample included forty amnesic MCI (multiple-domain: 22, single-domain: 18), and ten non-amnesic MCI (multiple-domain: 4, single-domain: 6). As control group, fifty healthy subjects were recruited in centers for retired people (31 males; age range: 59–87 years; mean age: 70 ± 6.7).

A cognitive screening by the Mini-Mental State Examination (corrected MMSE; AD: 18.33 ± 4.58, MCI: 25.98 ± 1.86, HC: 27.41 ± 1.63; $F_{2,147}$= 131.95, p<0.0001) was obtained for all participants. Major psychiatric diseases were excluded by assessing the State Trait Anxiety Index (*Y1*: AD: 37.02 ± 9.20, MCI: 36.90 ± 9.49, HC: 30.88 ± 5.90; $F_{2,141}$= 8.72, p=0.0003; *Y2*: AD: 38.68 ± 9.58, MCI: 39.66 ± 10.17.49, HC: 34.62 ± 8.86; $F_{2,141}$= 3.88, p=0.02) and the Hamilton Depression Rating Scale in all the groups (AD: 11.00 ± 5.74, MCI: 9.92 ± 5.07, HC: 8.12 ± 5.08; $F_{2,142}$= 3.62, p=0.03). Sleep quality was assessed by the Italian version of the Pittsburgh Sleep Quality Index (AD: 6.02 ± 3.74, MCI: 6.22 ± 3.60, HC: 6.56 ± 3.55; $F_{2,144}$= 0.27, p=0.76).

The diagnosis of AD or MCI for the clinical samples and the lack of cognitive impairment or dementia for the control group represented the inclusion criteria.

The exclusion criteria for all participants were: presence of neurological, psychiatric, or vascular disorders, diagnosed sleep disorders, previous history of seizures, the use of psychoactive and hypnotic drugs and history of alcoholism or drug abuse.

All participants gave written informed consent to participate in the study (also caregivers for AD patients). The study was performed in accordance with the declaration of Helsinki and was approved by the Local Ethics Committee of the Policlinico Universitario A. Gemelli – IRCCS and by the Institutional Review Board of the Department of Psychology of the University of Rome Sapienza.

Experimental design

The EEG electrodes montage procedure started at 19:00 h. At around 20:00 h, participants underwent 5 min of resting-state EEG recording (eyes-closed condition) in a video-monitored, soundproof and electrically shielded room. PSG-recording started according to the individual usual sleep schedule (21:00-23:00 h) and ended after the final spontaneous awakening. The morning resting-state EEG recording (5 min) started at least 1 h after the final awakening, to avoid possible confounding effects of sleep inertia (Ferrara et al., 2006; Gorgoni et al., 2015).

EEG recordings

EEG signals were recorded by a Micromed System (Micromed, Mogliano Veneto, Treviso, Italy) Morpheus digital polygraph, using Ag/AgCl electrodes. The electrode montages included 19 cortical sites (Fp1, F3, F7, C3, P3, T3, T5, O1, Fp2, F4, F8, C4, P4, T4, T6, O2, Fz, Cz, Pz, Oz), A1 and A2 at mastoids, 2 EOG, 2 submental EMG, ECG, and peripheral hemoglobin saturation. The EEG signals were referenced to the ground electrode at Fpz. Impedances were kept below 5

KΩ. Signals were recorded with a sampling frequency of 256 Hz, A/D conversion at 16 bit, a pre-amplifier amplitude range of ± 3.200 μV, pre-filters at 0.15 Hz and a notch filter (50 Hz).
EEG signals were off-line re-referenced to the average of the mastoids (A1-A2) and pass-band filtered between 0.33 Hz and 30 Hz (second order Bessel filter).

Quantitative EEG analyses

Sleep EEG analysis

Sleep recordings were scored offline by visual inspection (epoch duration: 20 s), according to the standard criteria (AASM, (Iber et al., 2007)), and the scoring was then revised by a second expert. The following macrostructural parameters were obtained: sleep onset latency (defined by the first appearance of a K-complex or a >0.5s sleep spindle); REM latency; sleep stages duration (%); wakefulness after sleep onset (WASO), in minutes; number of awakenings; total sleep time, defined as the sum of time spent in N1, N2, N3 and REM; total bed time; sleep efficiency index (total sleep time / total bed time x 100).
Quantitative EEG analyses were performed by Fast Fourier Transform (FFT) on the 20s artefact-free epochs (4 s periodogram), separately for NREM and REM sleep. Artefacts were visually detected and the whole 20-s epoch removed from the analysis. The average time period of sleep recordings after the artifacts rejection for the further analyses was 131 ± 49.3 min. Power spectra were computed in the 0.50 – 30 Hz range (bin resolution: 0.25 Hz) for each EEG channel and averaged across epochs for REM and NREM (merging N2 and N3 epochs) sleep, separately. Log-transformed spectral powers of adjacent bins were averaged to obtain the power for the following frequency bands: delta (0.50 – <5 Hz), theta (5.00 – <8 Hz), alpha (8.00 – <12 Hz), sigma (12.00 – <16 Hz), and beta (16.00 – <25 Hz).

Waking EEG analysis

Artifacts were detected by visual inspection with the consensus of two scorers and removed on a 2s-epoch basis. The power spectra of the remaining 2 s epochs (mean recording time: 62 ± 40.1 s) were computed in the 0.50 – 30 Hz range (bin resolution: 1 Hz, except for the 0.5–1 Hz) by FFT for each EEG channel and then averaged across the recorded period. The spectral power for the canonical frequency bands (delta: 0.5 – <5 Hz, theta: 5 – <8 Hz, alpha: 8 – <13 Hz, beta1: 13 – <16 HZ, beta2: 16 – <25 Hz) was obtained by averaging the log-transformed power ($\log_{10}$) across the corresponding bins.

Statistical analysis

EEG alterations in MCI and AD across the wake-sleep cycle

The macrostructural features of sleep and EEG power values during sleep (NREM, REM) and wakefulness [evening (PM), morning (AM)] have been compared between groups by one-way ANOVAs, separately for each channel and frequency band. For significant ANOVAs, the post hoc comparisons were performed by unpaired two-tailed t-tests.

Changes in the waking EEG after a night of sleep and the relationship with sleep EEG activity

Possible differences in the modulation of waking cortical activity by sleep-related homeostatic factors have been investigated by mixed-design ANOVAs *Group* (AD vs. MCI vs. HC) x *Time of day* (PM vs. AM) on EEG power values before and after sleep. Post hoc comparisons have been performed by two-tailed unpaired (between-groups comparisons) or paired (AM vs. PM comparisons) t-tests only on significant *Group* x *Time of day* interactions in the mixed-design ANOVAs.
The relationship between cortical activity during sleep and the amount of over-night changes in the waking EEG has been evaluated by correlating the EEG power changes [$\log_{10}$ (AM power) - $\log_{10}$ (PM power)] at the most representative cortical sites of the *Group* x *Time of day* interactions with the EEG power during sleep. In other words, the selected changes of waking EEG power have been correlated by Pearson's *r* with the EEG power of the whole topography in the different frequency bands of NREM and REM sleep, separately for each group.

The EEG slowing in REM sleep and wakefulness

In order to assess the relations between the EEG slowing during sleep and wakefulness, we considered the synthetic index of the ratio between slow- (delta + theta) and fast-frequency EEG activity [alpha + beta1 (sigma for sleep) + beta2 (beta for sleep)] for each cortical site. Previous studies found that such power ratio index in AD patients shows a strong negative correlation with normalized cerebral metabolic rate for oxygen assessed by PET (Buchan et al., 1997) and correlates with structural lesion assessed by computed tomography in patients with brain tumors (Nagata et al., 1985). These findings suggest that EEG slowing is closely associated with the reduction in cerebral oxygen metabolism and structural damage in the areas affected by the neurodegeneration process. This EEG slowing index has been compared between groups by one-way ANOVAs. The cortical site showing the most robust between-group difference during REM sleep has been correlated (Pearson's *r*) with the EEG slowing index of the whole topography in the wakefulness before and after sleep, separately for each group.

Correlation between EEG alterations and cognitive impairment

Finally, the EEG indexes that better highlight the alterations in MCI and AD in the different states of consciousness, as revealed by the between-group comparisons, have been correlated (Pearson's *r*) with the MMSE scores, as a measure of cognitive impairment.

The α-value of the comparisons between groups has been corrected by false discovery rate (FDR, (Benjamini and Hochberg, 1995)) computed on the p-values from the whole set of ANOVAs (722 comparisons including the number of derivations and frequency bands/composite index for all the assessed main factors and interactions; critic p= 0.0102).

Also, the α-value of the whole set of the correlation analyses has been corrected for multiple comparisons (1900 comparisons including the number of derivations and frequency bands/EEG indexes for the three groups on which the correlations have been computed) by FDR (critic p = 0.0054).

The sample size differs across specific analyses, because of the presence of missing data due to the poor quality of the EEG signals. Accordingly, the number of individuals in each group providing data for each analysis is reported in the corresponding results section.

Supplemental references

Benjamini, Y., Hochberg, Y., 1995. Controlling the False Discovery Rate: A Practical and Powerful Approach to Multiple Testing. J. R. Stat. Soc. Ser. B 57, 289–300.

Buchan, R.J., Nagata, K., Yokoyama, E., Langman, P., Yuya, H., Hirata, Y., Hatazawa, J., Kanno, I., 1997. Regional correlations between the EEG and oxygen metabolism in dementia of Alzheimer's type. Electroencephalogr. Clin. Neurophysiol. 103, 409–417. https://doi.org/10.1016/S0013-4694(97)00015-5

Carlesimo, G.A., Caltagirone, C., Gainotti, G., 1996. The Mental Deterioration Battery: normative data, diagnostic reliability and qualitative analyses of cognitive impairment. The Group for the Standardization of the Mental Deterioration Battery. Eur. Neurol. 36, 378–84. https://doi.org/10.1159/000117297

Chandler, M.J., Lacritz, L.H., Cicerello, A.R., Chapman, S.B., Honig, L.S., Weiner, M.F., Cullum, C.M., 2004. Three-word recall in normal aging. J. Clin. Exp. Neuropsychol. 26, 1128–33. https://doi.org/10.1080/13803390490515540

Ferrara, M., Curcio, G., Fratello, F., Moroni, F., Marzano, C., Pellicciari, M.C., De Gennaro, L., 2006. The electroencephalographic substratum of the awakening. Behav. Brain Res. 167, 237–244. https://doi.org/10.1016/j.bbr.2005.09.012

Gorgoni, M., Ferlazzo, F., D'Atri, A., Lauri, G., Ferrara, M., Rossini, P.M., De Gennaro, L., 2015. The assessment of somatosensory cortex plasticity during sleep deprivation by paired associative stimulation. Arch. Ital. Biol. https://doi.org/10.12871/000398292015236

Iber, C., Ancoli-Israel, S., Chesson, A., Quan, S., 2007. The AASM Manual for the Scoring of

Sleep and Associates Events: Rules, Terminology and Technical Specifications, First. ed, American Academy of Sleep Medicine. Westchester, IL.

McKhann, G.M., Knopman, D.S., Chertkow, H., Hyman, B.T., Jack, C.R., Kawas, C.H., Klunk, W.E., Koroshetz, W.J., Manly, J.J., Mayeux, R., Mohs, R.C., Morris, J.C., Rossor, M.N., Scheltens, P., Carrillo, M.C., Thies, B., Weintraub, S., Phelps, C.H., 2011. The diagnosis of dementia due to Alzheimer's disease: Recommendations from the National Institute on Aging-Alzheimer's Association workgroups on diagnostic guidelines for Alzheimer's disease. Alzheimer's Dement. 7, 263–269. https://doi.org/10.1016/j.jalz.2011.03.005

Nagata, K., Gross, C.E., Kindt, G.W., Geier, J.M., Adey, G.R., 1985. Topographic Electroencephalographic Study with Power Ratio Index Mapping in Patients with Malignant Brain Tumors. Neurosurgery 17, 613–619. https://doi.org/10.1227/00006123-198510000-00014

Petersen, R.C., Doody, R., Kurz, A., Mohs, R.C., Morris, J.C., Rabins, P. V., Ritchie, K., Rossor, M., Thal, L., Winblad, B., 2001. Current Concepts in Mild Cognitive Impairment. Arch. Neurol. 58, 1985. https://doi.org/10.1001/archneur.58.12.1985

Portet, F., 2006. Mild cognitive impairment (MCI) in medical practice: a critical review of the concept and new diagnostic procedure. Report of the MCI Working Group of the European Consortium on Alzheimer's Disease. J. Neurol. Neurosurg. Psychiatry 77, 714–718. https://doi.org/10.1136/jnnp.2005.085332

Rey, A., 1983. Reattivo della figura complessa: manuale. Organizzazioni speciali.

Spinnler, H., Tognoni, G., dell'invecchiamento, G. italiano per lo studio neuropsicologico, 1987. Standardizzazione e taratura italiana di test neuropsicologic, Italian journal of neurological sciences: Supplementum. Masson Italia Periodici.

Zaudig, M., 1992. A New Systematic Method of Measurement and Diagnosis of "Mild Cognitive Impairment" and Dementia According to ICD-10 and DSM-III-R Criteria. Int. Psychogeriatrics 4, 203–219. https://doi.org/10.1017/S1041610292001273
